# Mesenchymal Transglutaminase 2 Activates Epithelial ADAM17: Link to G-Protein-Coupled Receptor 56 (ADGRG1) Signalling

**DOI:** 10.3390/ijms25042329

**Published:** 2024-02-16

**Authors:** Lea Bauer, Jessica Edwards, Andreas Heil, Sharon Dewitt, Heike Biebermann, Daniel Aeschlimann, Vera Knäuper

**Affiliations:** 1 College of Biomedical and Life Sciences, School of Dentistry, Cardiff University, Cardiff CF14 4XY, UKdewitt@cardiff.ac.uk (S.D.); 2 Institute of Experimental Pediatric Endocrinology, Corporate Member of Freie Universität Berlin and Humboldt-Universität zu Berlin, Charité—Universitätsmedizin Berlin, Augustenburger Platz 1, 13353 Berlin, Germany

**Keywords:** ADAM17, transglutaminase 2, adhesion G-protein-coupled receptor, GPR56, ADGRG1, EGFR-ligand

## Abstract

A wound healing model was developed to elucidate the role of mesenchymal-matrix-associated transglutaminase 2 (TG2) in keratinocyte re-epithelialisation. TG2 drives keratinocyte migratory responses by activation of disintegrin and metalloproteinase 17 (ADAM17). We demonstrate that epidermal growth factor (EGF) receptor ligand shedding leads to EGFR-transactivation and subsequent rapid keratinocyte migration on TG2-positive ECM. In contrast, keratinocyte migration was impaired in TG2 null conditions. We show that keratinocytes express the adhesion G-protein-coupled receptor, ADGRG1 (GPR56), which has been proposed as a TG2 receptor. Using ADAM17 activation as a readout and luciferase reporter assays, we demonstrate that TG2 activates GPR56. GPR56 activation by TG2 reached the same level as observed with an agonistic N-GPR56 antibody. The N-terminal GPR56 domain is required for TG2-regulated signalling response, as the constitutively active C-GPR56 receptor was not activated by TG2. Signalling required the C-terminal TG2 β-barrel domains and involved RhoA-associated protein kinase (ROCK) and ADAM17 activation, which was blocked by specific inhibitors. Cell surface binding of TG2 to the N-terminal GPR56 domain is rapid and is associated with TG2 and GPR56 endocytosis. TG2 and GPR56 represent a ligand receptor pair causing RhoA and EGFR transactivation. Furthermore, we determined a binding constant for the interaction of human TG2 with N-GPR56 and show for the first time that only the calcium-enabled “open” TG2 conformation associates with N-GPR56.

## 1. Introduction

Wound healing is a complex biological process, and impairments manifest clinically as diverse problems, ranging from defective extracellular matrix (ECM) remodelling to failure of wound re-epithelialisation or skin barrier formation [1,2]. Re-epithelialisation requires disintegrin and metalloproteinase 17 (ADAM17)-dependent release of epidermal growth factor receptor (EGFR)-ligands, activating EGFR signalling in the epithelium, and is regulated by a functional interplay of the epithelial layer with the underlying ECM [3]. EGFR signalling is essential for epithelial migration, suppression of chronic inflammatory signalling, as well as functional skin barrier formation in vivo, as demonstrated in conditional null mice targeting ADAM17 or EGFR in keratinocytes [4]. We showed that transglutaminase 2 (TG2) levels modulate the dermal fibroblast migration through regulation of focal adhesion turnover and MMP2 activation, as well as matrix assembly and wound contraction through ECM crosslinking [5]. TG2 null mice have delayed wound healing in vivo [6,7,8]. However, the molecular mechanism is still unclear, and could in part relate to an altered innate immune response observed in tissue injury models in vivo [6,7,9,10]. To selectively assess whether ECM changes associated with TG2 deficiency impact keratinocyte responses via mesenchymal epithelial cross-talk, we developed a re-epithelialisation model and employed it here to assess the role of TG2 in cellular cross-talk and to dissect the relevant signalling events.

The adhesion GPCR, ADGRG1 (GPR56), has been identified as a TG2 interaction partner in cancer invasion [11,12]. Developmentally, GPR56 regulates neural progenitor migration in the brain frontal cortex, with loss of function mutations (for example, R_565_W) causing bilateral frontoparietal polymicrogyria (BFPP) [13,14,15,16]. GPR56 has also been implicated in axonal myelination in the central and peripheral nervous system, where it regulates oligodendrocyte development in the former, and radial sorting of axons and myelin maintenance in the latter [17,18]. Mechanistically, this appears to be consistently linked to RhoA activation and Gα_12/13_ association [17,19,20]. GPR56 couples to G_q_ and Gα_12/13_, and the TG2 and type III collagen were identified as potential ligands [11,21]. Interestingly, nervous system development appears to involve GPR56-collagen III interaction, whereas microglial TG2 has recently been shown to drive myelination and myelin repair via GPR56 in oligodendrocyte precursor cells in the presence of laminin [22]. GPR56 is auto proteolytically processed at the G-protein-coupled receptor proteolysis site (GPS), localized in the autoproteolysis inducing (GAIN) domain into an N-terminal and C-terminal fragment required for trafficking and functional folding [14,15,23,24]. GPR56 is expressed in many ectodermally derived cells, and hence expression is widespread. The role of GPR56 in cancer is complex, with high expression in aggressive glioblastoma [25] and loss of GPR56 expression in metastatic melanoma [11].

TG2 is a transglutaminase family member of structurally and functionally related enzymes that stabilize protein assemblies through intra- or intermolecular γ-glutamyl-ε-lysine crosslinks [26,27,28]. Extracellular TG2 not only crosslinks ECM and promotes cell-ECM interactions [5] but can also directly regulate receptor clustering and activity [29]. TG2 has GTP binding and hydrolysis ability, enabling it to act as an intracellular G protein. Cell surface localisation of TG2 leads to RhoA activation [30], a function shared with GPR56. The intra- and extracellular TG2 functions depend on mutually exclusive conformations that are specific to the environment, oxidation, Ca^2+^, or GTP binding status [6,26,31,32]. Different TG2 conformations contribute to numerous TG2 functions, and TG2 deficiency impacts both wound healing and cancer development [33,34,35,36].

We show the expression of GPR56 in primary and immortalized keratinocytes, and demonstrate the activation of ADAM17 by TG2 in GPR56-expressing cells, which is functionally linked to RhoA activation and validates our findings using luciferase reporter assays. Analysis of C-GPR56, a mutant lacking the N-terminal TG2 binding domain, confirmed that the N-terminal GPR56 domain is required for receptor activation in response to TG2 treatment. Extracellular TG2 binds to cell surface GPR56, and is internalised in a GPR56-dependent manner, leading to partial co-localisation with N-terminal GPR56 in endocytic vesicles, a behaviour reminiscent of ligand-receptor pairs.

## 2. Results

### 2.1. TG2-Positive ECM Facilitates Keratinocyte Re-Epithelialisation Response through ADAM17-Dependent EGFR Transactivation

**Figure 1:**
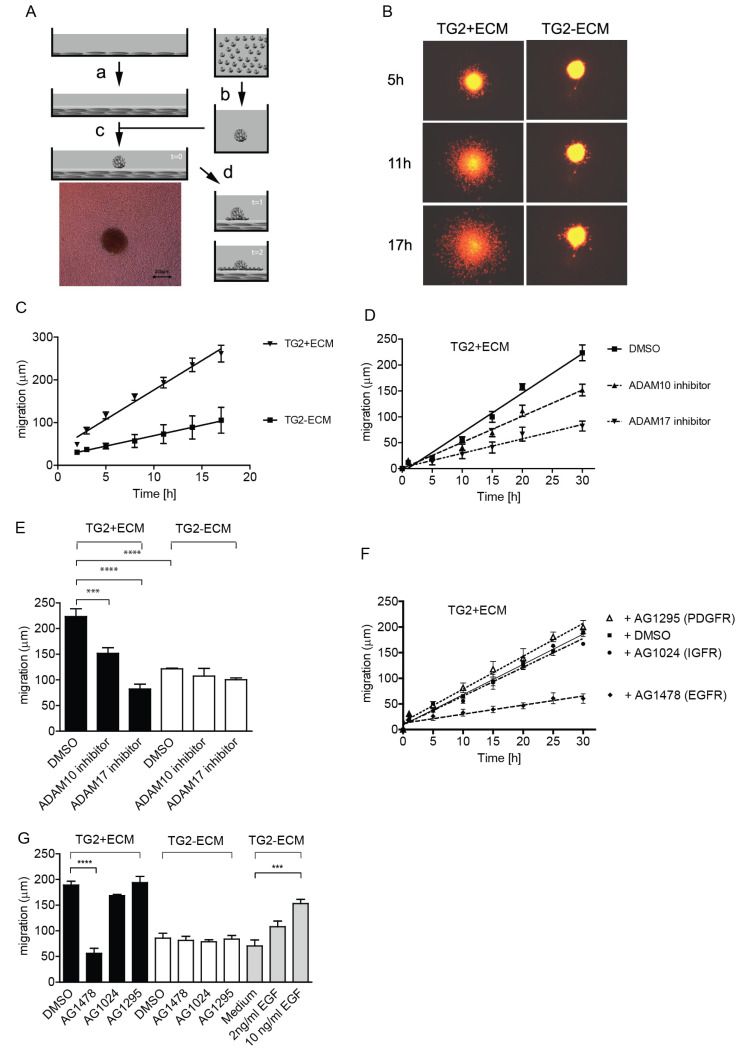
Mesenchymal TG2 enhances keratinocyte migration in a wound healing model in an ADAM17- and EGFR-dependent manner. (**A**) Schematic representation of re-epithelialization model. A confluent fibroblast layer was grown for 10 days to establish a dermal tissue-like 3D ECM (a). Keratinocytes (2.5 × 10^3^ cells) were labelled with a cell-tracker dye and kept in a hydrophobic environment for 20 h to form a spheroid (b). The spheroid was placed onto fibroblast ECM shown in (c) with a phase contrast micrograph below. Keratinocyte migration was followed by epifluorescence timelapse microscopy as indicated in (d). (**B**) Micrographs of fluorescently labelled n-tert 1 keratinocyte spheroids at different time points, following placing onto ECM established with wild type HCA2 fibroblasts (TG2+ ECM) or HCA2 fibroblasts deficient in TG2 (TG2− ECM). (**C**) Keratinocyte migration quantified by identifying outer boundaries at each time point using a rolling ball algorithm and calculating average expansion distance from the integrated surface area (for details see Supplement Figure S1A). Comparison of keratinocyte migration on TG2+ ECM and TG2− ECM in FAD medium (+/−s.e.m., *n* = 5). (**D**) Keratinocyte migration on TG2+ ECM is partially inhibited by 10μM ADAM10 inhibitor, and completely by 10μM ADAM17 inhibitor, when compared to DMSO control (+/−s.e.m., *n* = 6). (**E**) Comparison of keratinocyte expansion on TG2+ ECM (black bars) or TG2− ECM (white bars) after 30 h (+/−s.e.m., *n* = 6) shows that only migration on TG2+ ECM is ADAM dependent (Anova with Tukey’s post-test, *p* < 0.0005 for ADAM10 inhibitor and *p* < 0.0001 for ADAM17 inhibitor). (**F**) Time course of keratinocyte migration on TG2+ ECM in the presence of 10 μM receptor kinase inhibitor blocking EGFR, IGFR, or PDGFR, compared to DMSO control in 1% serum FAD medium without EGF and insulin (+/−s.e.m., *n* = 5). EGFR-inhibitor blocked migration. (**G**) Comparison of keratinocyte expansion on TG2+ ECM (black bars) or TG2− ECM (white bars) at 30 h in the presence of EGFR, IGFR, or PDGFR inhibitors shows inhibition by the EGFR inhibitor only on TG2+ ECM (Anova, *p* < 0.0001). Supplementation of medium with EGF enhances keratinocyte migration on TG2− ECM (grey bars) significantly (Anova, *p* = 0.0006). All data are average +/−s.e.m. (*n* = 5)). *p* values of <0.001 equals (***), and *p* values of <0.0001 equals (****).

To study mesenchymal-epithelial cross-talk, we developed a 3D wound healing model where a multicellular, quiescent, fluorescently labelled keratinocyte spheroid is placed on a dermal-like 3D ECM, as outlined schematically (Figure 1A). We employed the N-tert1-immortalized keratinocyte cell line, which is fully competent to differentiate in organotypic cultures [37], to analyse re-epithelialisation responses to devitalized dermal-like ECM (Figure S1B) produced by TG2-positive (TG2+) or negative (TG2−) human fibroblasts [5]. Keratinocyte spheroid expansion was monitored using timelapse microscopy. TG2+ ECM supported rapid keratinocyte migration, whereas very little migration was evident on TG2− ECM (Figure 1B). Radial expansion of the keratinocyte spheroid was quantified using the algorithm, detailed in Supplementary Figure S1A, to avoid observer bias. Keratinocyte migration on TG2+ ECM was significantly increased (Figure 1C). We then investigated whether metalloproteinase activity was required for increased migration on TG2+ ECM, and showed partial inhibition by GM6001, TAPI-1 (Figure S1C), or ADAM10 inhibitor (Figure 1D). Only ADAM17 inhibition was effective in blocking migration on TG2+ ECM, reducing it to the level seen with TG2− ECM (Figure 1D). Comparison of keratinocyte migration on TG2+ ECM versus TG2− ECM in the presence and absence of ADAM inhibitor showed that only migration on TG2+ ECM was ADAM17 dependent. Migration on TG2− ECM was ADAM independent (Figure 1E). This indicates that TG2+ ECM activates ADAM17 in keratinocytes leading to enhanced motility. We hypothesized that this was likely linked to transactivation of EGFR. However, as insulin-like growth factor receptor (IGFR) modulates integrin-mediated motility [38] and a TG2–integrin complex promotes the platelet-derived growth factor receptor (PDGFR) signalling [39], we added specific kinase inhibitors to the medium to assess their contribution to keratinocyte migration. Only the EGFR inhibitor AG1478 was efficient at blocking keratinocyte migration on TG2+ ECM, while PDGFR and IGFR inhibitors were unable to block migration (Figure 1F,G, black bars). None of the kinase inhibitors affected migration on TG2− ECM (Figure 1G, white bars), but the addition of the EGFR-ligand, such as EGF, rescued impaired keratinocyte migration on TG2− ECM (Figure 1G, EGF addition, grey bars).

### 2.2. TG2 Promotes Keratinocyte Proliferation through ADAM17 Dependent EGFR-Ligand Shedding

**Figure 2:**
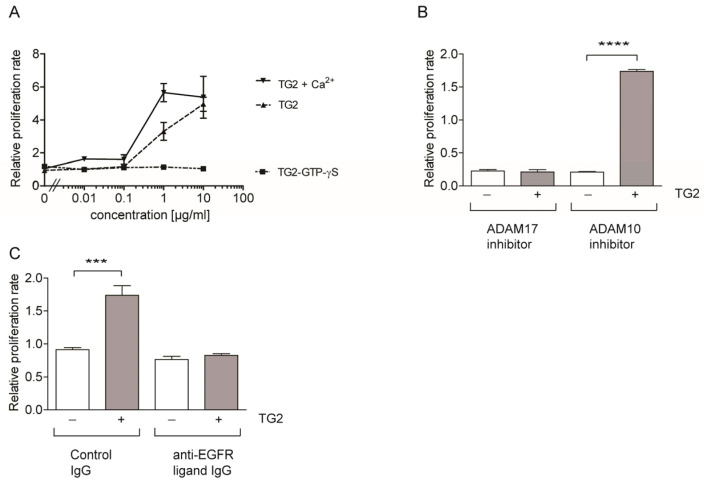
Recombinant TG2 activates EGFR signalling in keratinocytes. (**A**) n-tert 1 keratinocytes (5 × 10^3^/well) were seeded into 24-well plates and left to spread for 6 h prior to switching to 1% serum FAD medium, lacking EGF. Cells were stimulated with recombinant human TG2, Ca^2+^ activated TG2, or TG2-GTPγS (or carrier control) at the concentration indicated. After 24 h and 72 h, the cell number was determined from the amount of MTT converted into formazan. Data are the mean relative increase in cell number (±s.e.m. triplicates) over control of a representative experiment (*n* = 3). (**B**) Keratinocytes were treated with 10 μM ADAM10 or ADAM17 inhibitor or DMSO, and either left untreated or stimulated with 10 μg/mL TG2. Proliferation rate relative to unstimulated DMSO control was assessed and is given as mean ±s.e.m. (*n* = 3). ADAM17 inhibitor, but not ADAM10 inhibitor, blocked TG2-mediated stimulation of cell proliferation (Anova with Tukey’s post-test, *p* < 0.0001 for ADAM10 inhibitor and *p* = 0.96 for ADAM17 inhibitor). (**C**) Keratinocytes were stimulated with 10 μg/mL TG2 where indicated, either in the absence or presence of 10 μg/mL each of anti-EGF, anti-HB-EGF, and the anti-TGFα antibodies or 30 µg/mL control antibody and the change in proliferation rate were assessed. (*n* = 3). TG2-stimulated proliferation only in the absence of inhibitory antibodies (Anova with Tukey’s post-test, *p* = 0.0004 versus *p* = 0.94)). *p* values of <0.001 equals (***), and *p* values of <0.0001 equals (****).

Having established that TG2+ ECM caused EGFR transactivation in keratinocytes, we wanted to establish whether this response was due to TG2 itself, as opposed to changes in ECM composition and/or crosslinking thereof. As EGF is a potent keratinocyte mitogen, we performed keratinocyte proliferation experiments by adding human recombinant TG2, Ca^2+^ activated TG2, or TG2-GTPγS complex to keratinocytes in 1% FCS medium to minimize background proliferation due to serum-derived EGF. In the presence of TG2 or Ca^2+^ activated TG2, but not the TG2-GTPγS complex, a dose-dependent increase in keratinocyte proliferation was observed while the TG2-GTPγS complex was inactive (Figure 2A). We then investigated whether the TG2 response was dependent on ADAM activity. Keratinocyte proliferation was blocked by ADAM17 inhibitor, but not by ADAM10 inhibitor (Figure 2B), in line with migration data on TG2+ ECM (Figure 1D). To address which EGFR-ligands were involved in EGFR-transactivation, we employed inhibitory antibodies. Figure 2C shows that the combination of three inhibitory antibodies targeting EGF, TGFα, and HB-EGF was effective in blocking the proliferation response to TG2. IgG control antibody treatment had no effect, whereas single inhibitory antibody treatment only partially blocked TG2-mediated proliferation (Supplementary Figure S4B). Collectively, our data suggest that keratinocytes express an unknown receptor for TG2, and that the interaction between this receptor and TG2 was causing transactivation of EGFR signalling through ADAM17 activation.

GPR56 was identified as a TG2 binding partner [11], which led us to hypothesize that GPR56 may be the TG2 receptor responsible for EGFR transactivation in keratinocytes. GPR56 mRNA expression in our N-tert1-immortalized keratinocytes, as well as in primary keratinocytes, was confirmed by PCR, with increased expression levels during keratinocyte differentiation (Supplementary Figure S2A,B). Western blot analysis showed positive staining for the N-GPR56 fragment in N-tert1 keratinocyte lysates (Figure S2C).

### 2.3. GPR56 Signaling Regulates ADAM17 Activity

**Figure 3:**
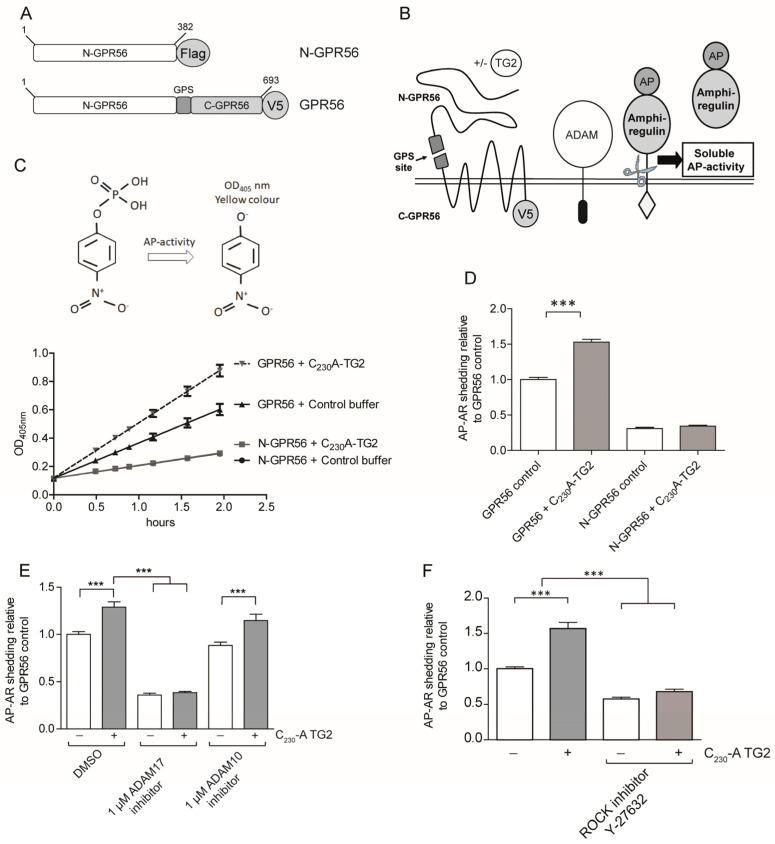
GPR56 activates ADAM17 and Rho kinase. (**A**) Expression constructs used for GPR56 signalling. (**B**) Schematic representation of shedding assay principle: GPR56-mediated activation of ADAM17 leads to release of the ectodomain of alkaline phosphatase tagged amphiregulin (AP-AR-ECD). (**C**) HEK293 cells co-expressing AP-AR and either GPR56 or N-GPR56 were stimulated for 1 h with 20 µg/mL C_230_A TG2 or buffer control, followed by analysis of conditioned medium for AP activity by measuring 4-NPP hydrolysis. The reaction of substrate hydrolysis is shown at the top of (**C**) leading to the generation of 4-nitrophenol by soluble AP-AR. This is measured at OD_405 nm_ over time (data from a representative experiment with 4 replicas). (**D**) The 4-NPP hydrolysis rate of buffer treated GPR56 transfected cells was set to 1, and at least 3 independent experiments with 4 internal repeats were analysed using Anova with post-Tukey analysis. Data are mean +/−s.e.m., and indicate a significant increase in AP-AR release into medium upon TG2 treatment in GPR56 but not N-GPR56 expressing cells. (**E**) Cells were serum starved in the presence of ADAM10 or ADAM17 inhibitor or carrier control for 1 h prior to analysis of AP-AR shedding in the presence or absence of inhibitors (*n* = 3). (**F**) AP-AR shedding was analysed in the presence of 5 µM ROCK inhibitor (*n* = 3). *p* values of <0.001 equals (***).

Although the literature evidence shows that TG2 binds to the N-terminus of GPR56 [11], it was unclear whether this causes a signalling response. In order to show directly that GPR56 was able to activate ADAM17-dependent EGFR-ligand shedding, we required a system that was amenable to experimental manipulation of GPR56 functionality. Therefore, we co-expressed the human GPR56 cDNA together with an alkaline phosphatase tagged amphiregulin cDNA (AP-AR) in HEK293 cells. As a control, we co-expressed N-GPR56 that is truncated at the GPS site and signalling-incompetent (Figure 3A). The assay principle is shown in Figure 3B, and both GPR56 and N-GPR56 were expressed at least in part at the cell surface (Supplementary Figure S3). We noted that the co-expression of intact GPR56 with AP-AR led to a significant increase in shed AP activity, in contrast to the co-expression of AP-AR with signalling-incompetent N-GPR56 (Figure 3C,D compare GPR56 control with N-GPR56 control). Partial GPR56 activation in the absence of exogenous ligands has been reported by others [20,40,41], and is linked to active receptor conformations upon receptor over-expression. We then treated GPR56 or N-GPR56 and AP-AR co-transfected cells with native TG2 or an oxidation resistant mutant of TG2 (C_230_A TG2). TG2 harbours a redox-sensitive triad of cysteine residues whereby Cys_230_ promotes the formation of a Cys_370_-Cys_371_ disulfide bond and consequential enzyme inactivation [32]. Given that cell responses in keratinocytes were dependent on enzyme conformation (Figure 2A), we generated the oxidation resistant C_230_A TG2 mutant, and included this in our cell-based experimentation to prevent any premature enzyme inactivation. We saw an increase in released AP-AR ectodomain (AP-AR-ECD) only in the presence of full length GPR56 (Figure 3C,D; for native TG2 see below), suggesting ligand-dependent signalling that requires an intact receptor. C_230_A TG2 (or native TG2) was employed at the 20 µg/mL optimal dose to ensure maximal enzyme activity during stimulation, as active enzyme, but not TG2-GTPγS complex, stimulated keratinocytes (Figure 2A). To confirm that EGFR-ligand release was dependent on ADAM17 activity, GPR56 and AP-AR co-transfected cells were treated with ADAM17 or ADAM10 inhibitor and compared to DMSO solvent control. AP-AR shedding was monitored in response to buffer or TG2 treatment. Figure 3E shows that only the ADAM17 inhibitor ablated GPR56 dependent AP-AR-ECD release in both unstimulated and stimulated conditions. In order to verify that an intracellular signalling response was regulating ADAM17 activity, we tested whether this was linked to ROCK activity. A link between GPR56 and RhoA signalling has been proposed [19,20]. ROCK inhibition with Y-27632 blocked AP-AR shedding in non-stimulated, as well as TG2-stimulated conditions (Figure 3F). These data indicate that TG2 stimulates RhoA activity and ADAM17 activation in a GPR56 dependent manner.

### 2.4. TG2 Treatment Does Not Induce Detectable Intermolecular Crosslinking of GPR56

**Figure 4:**
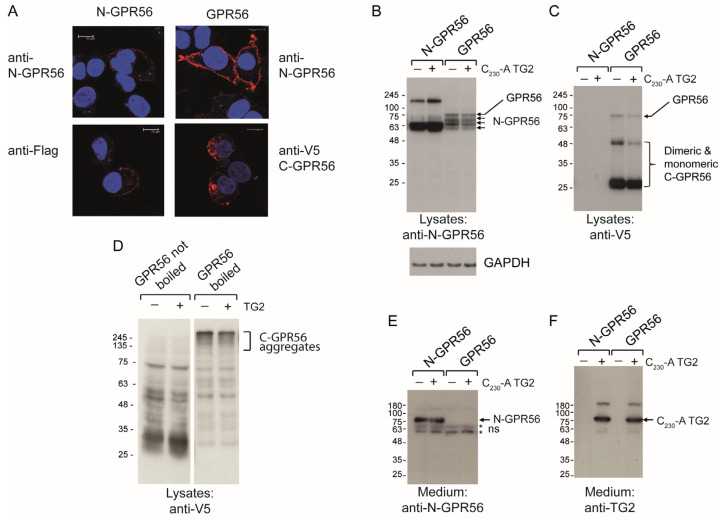
TG2 treatment of GPR56 or N-GPR56-expressing cells does not result in detectable intermolecular crosslinking of the N-terminal GPR56 domain. (**A**) Optical section acquired by Confocal microscopy showing N-GPR56 and GPR56 expression in transfected cells under non permeabilising conditions (Top panel: anti N-GPR56 antibody with GPR56 signals in red) and permeabilising conditions (Bottom panel: anti Flag antibody for N-GPR56-expressing cells and anti-V5 antibody for GPR56-expressing cells shown in red). Scale bar 10 µm. (**B**,**C**) Western blot analysis of cell lysate from AP-AR shedding experiments represented in Figure 3D stained for the N-terminal GPR56 domain and a GAPDH loading control (**B**) or the V5 epitope tag recognizing C-terminal GPR56 (**C**). Note that N-GPR56 is not recognized by the V5 antibody. (**D**) Detection of C-terminal GPR56 fragments required that samples were incubated in SDS sample buffer without heat treatment to avoid loss through aggregation/precipitation. (**E**,**F**) Western blot analysis of conditioned medium from AP-AR shedding experiment represented in Figure 3D stained with anti-N-GPR56 antibody or CUB7402 anti-TG2 antibody. Non-specific bands in medium are indicated by asterisks.

GPR56 cell surface levels were analysed using confocal microscopy. Non permeabilised cells stained with N-GPR56 antibody and Alexa-568-conjugated secondary antibodies showed abundant cell surface staining in GPR56 expressing cells, but also some weak cell surface staining of N-GPR56 expressing cells (Figure 4A top panels and Figure S3B), suggesting that N-GPR56 interacts not only with C-GPR56, but also weakly with other cell surface proteins. Staining of permeabilised cells with anti-Flag antibody detected some surface N-GPR56-Flag, as well as intracellular staining. Staining with anti-V5 antibody (C-GPR56) identified cell surface and intracellular staining for GPR56, confirming surface localization of the respective proteins (Figure 4A, bottom panels). Note, staining of lysates with anti-V5 antibody detected main bands at 27, 50, and 75 kDa of GPR56 expressing cells, whereby the 75 kDa band corresponds to unprocessed, full length GPR56 (Figure 4C).

As receptor clustering might elicit a signalling response, we analysed cell lysates and medium from shedding experiments for evidence of TG2-dependent, intermolecular crosslinking of the N-terminal GPR56 domain involved in TG2 binding by Western blotting, both for cells expressing full length receptor or signalling incompetent N-GPR56. In lysates from GPR56-transfected cells, N-GPR56 antibody detected 4 distinct bands at 75, 70, 65, and 60 kDa (Figure 4B), in good agreement with previous reports [20,24]. C_230_-A TG2 treatment did not influence the banding pattern for N-GPR56, indicating the lack of intermolecular crosslinking, or these were below the detection limit (Figure 4B). Irrespective of C_230_A TG2 treatment, lysates from N-GPR56-transfected cells showed a strong broad 60–68 kDa band and a less abundant high molecular weight component at 180 kDa (Figure 4B). The latter band only occurred when using non-boiling conditions for SDS-PAGE, and hence does not represent a covalent crosslinking product. C-GPR56 fragments were only detectable using non-boiling SDS-PAGE conditions, as they formed aggregates when boiled (Figure 4D). Medium contained specific bands for N-GPR56 in N-GPR56-transfected cells only, indicating that the N-GPR56 domain remained cell-associated in full length GPR56-expressing cells (Figure 4E). Conditioned medium probed for TG2 showed staining in samples supplemented with C_230_-A TG2, with a major band at 80 kDa and minor bands at ~180 and 55 kDa (Figure 4F). The additional bands likely correspond to crosslinked TG2 dimers and a TG2 fragment lacking β-barrel domains. Therefore, while some autocatalytic crosslinking of TG2 was evident, intermolecular crosslinking of GPR56 alone or a TG2-GPR56 complex was not detected.

### 2.5. GPR56 Activation by TG2 or Agonistic N-GPR56 Antibody Requires N-Terminal GPR56 as Determined Using Shedding or Rho-Luciferase Reporter Assays

**Figure 5:**
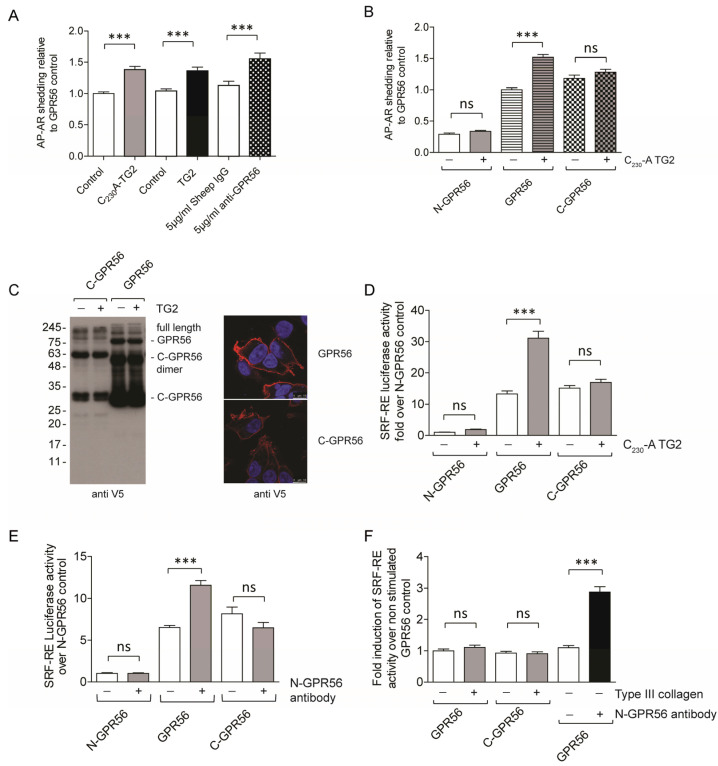
GPR56 activation by TG2 and anti-N-GPR56 antibody requires full length GPR56. (**A**) Comparison of C_230_-A TG2 (20 µg/mL), TG2 (20 µg/mL) and anti-N-GPR56 antibody (5 µg/mL) for their ability to activate full-length GPR56-dependent AP-AR shedding. (**B**) GPR56-dependent activation of shedding by TG2 requires intact GPR56. C-GPR56 is auto-active and not activated by TG2 (*n* = 3). (**C**) Western blot panel: Detection of the C-terminal GPR56 fragment in GPR56 and C-GPR56 expressing cells using the V5-epitope antibody. Confocal microscopy panel: Cell surface expression of GPR56 and C-GPR56 using anti-V5 antibody in red. Scale bar 10µm. (**D**) Luciferase reporter assay to assess TG2 dependent signalling in N-GPR56, GPR56, and C-GPR56-expressing cells. To determine Rho activation, cells were co-transfected with N-GPR56, GPR56, or C-GPR56 plasmid and SRF-RE luciferase reporter. After 48 h post-transfection, cells were stimulated with 20 µg/mL C_230_A TG2 for 6 h, and luciferase activity was determined in lysates. TG2 activated GPR56 dependent SRF-RE luciferase reporter activity, while C-GPR56 was not stimulated (*n* = 3). (**E**) N-GPR56 antibody (5 µg/mL) activates GPR56 dependent SRF-RE luciferase reporter activity. Neither N-GPR56 nor C-GPR56 was stimulated by N-GPR56 antibody treatment. Controls were treated with control sheep IgG (*n* = 3). (**F**) Type III collagen (83 nM) did not activate GPR56. N-GPR56 antibody was used as a positive control (*n* = 3). *p* values of <0.001 equals (***).

We next compared wild type TG2 and N-terminal GPR56 antibody for their ability to induce AP-AR shedding in GPR56-expressing cells. Figure 5A demonstrates that TG2, as well as the N-terminal GPR56 antibody induced AP-AR shedding, indicating a specific signalling response caused by interactions of TG2 or N-GPR56 antibody with the N-terminal domain of GPR56. To substantiate this conclusion, we generated an auto active, C-terminal GPR56 expression vector encoding a truncated protein starting at the GPS site containing the 7TM domain [20] (See Figure S2D). C-GPR56 expression alone caused AP-AR shedding, but C-GPR56 was inert to TG2 stimulation (Figure 5B). Both GPR56 and C-GPR56 were expressed abundantly, as indicated by Western blot analysis of cell lysates (Figure 5C, Western blot panel), and showed cell surface expression by confocal microscopy using the anti-V5 antibody recognizing the C-terminal end of GPR56 (Figure 5C, Confocal panel).

To substantiate these findings a luciferase reporter assay that measures Rho kinase activity was used to investigate GPR56 activation by potential ligands to provide support for our finding that Rho activity was required for ADAM17 dependent shedding of AP-AR. Both, TG2 and agonistic N-GPR56 antibody stimulated luciferase activity in cells co-expressing SRF-RE reporter plasmid and GPR56, when compared to control buffer or control antibody treatment, respectively (Figure 5D,E). In contrast, there was no stimulation of luciferase activity in cells co-expressing N-GPR56 or C-GPR56 and SRF-RE reporter plasmids upon ligand stimulation over controls (Figure 5D,E). This confirms GPR56 dependent signalling in response to TG2 for the first time and shows that the N-terminal domain is required for ligand regulation of GPR56. We also tested type III collagen, a potential ligand of GPR56 [21] using the SRF-RE reporter assay which failed to induce a response, although N-GPR56 antibody, the positive control showed stimulation (Figure 5F).

### 2.6. TG2-Dependent GPR56 Signaling Requires C-Terminal TG2 β-Barrels, but Is Independent of GPR56 Tail Phosphorylation Sites

**Figure 6:**
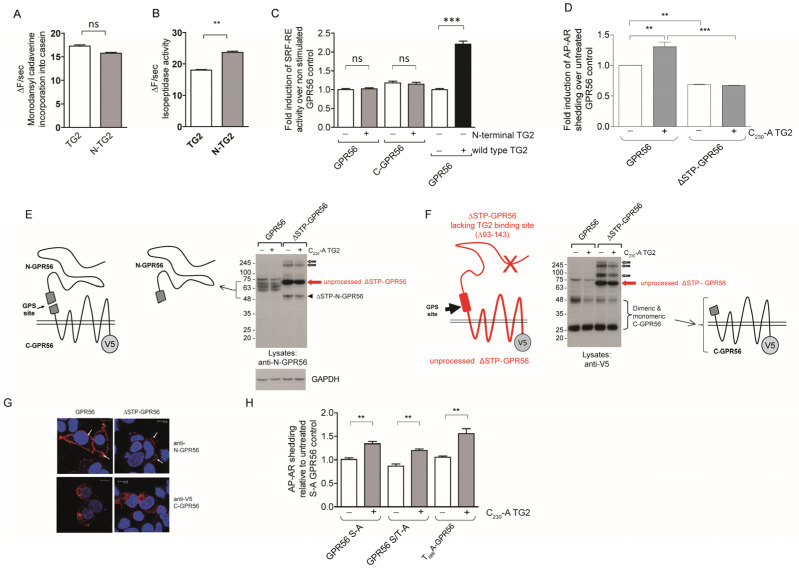
GPR56 activation requires N-terminal GPR56, C-terminal TG2, and is independent of C-terminal GPR56 phosphorylation sites. (**A**,**B**) Real-time measurements were conducted whereby the enzymatic reaction was initiated by Ca^2+^ injection (*n* = 3). Fluorescence in the absence of Ca^2+^ activation was subtracted from the fluorescence obtained with activated enzyme to correct for sample bleaching over time. Only the linear part of the fluorescence increase was used, and rates were determined by linear regression. We compared transamidation and isopeptidase activities of wild type TG2 (20 µg/mL) and N-TG2 lacking the β-barrel domains (20 µg/mL), showing that N-TG2 is catalytically functional. (**C**) N-TG2 does not activate GPR56- or C-GPR56-dependent Rho activity using SRF-RE reporter assays (*n* = 3). (**D**) ΔSTP-GPR56 lacking the TG2 binding site cannot be activated by TG2 using the AP-AR shedding assay (*n* = 3). (**E**,**F**) Western blot analysis of lysates from ΔSTP-GPR56 cells shows that it is only partially processed at the GPS-site (large arrow), and N-terminal as well as C-terminal domain aggregates are apparent (small arrows). A diagram of ΔSTP-GPR56 lacking the TG2 binding site is shown for clarity. (**G**) ΔSTP-GPR56 shows little cell surface expression when compared to GPR56 by confocal microscopy. Red stain corresponds to GPR56 staining using either anti-N-GPR56 or anti-V5 antibody. Blue nuclear stain. Scale bar 10µm. (**H**) GPR56 mutants lacking C-terminal Serine or Threonine tail phosphorylation sites are activated by TG2 using shedding assays (*n* = 3). *p* values of <0.01 equals (**), *p* values of <0.001 equals (***).

We then tested whether the β-barrel domains of TG2 were required for signalling. TG2 lacking the β-barrel domains (N-TG2) displayed comparable activity to native TG2 in isopeptidase and amine incorporation assays, suggesting N-TG2 is active (Figure 6A,B). We then tested N-TG2 for its ability to activate GPR56-dependent SRF-RE luciferase activity. N-TG2 was inactive, while wild type TG2-activated GPR56 in the same experiment (Figure 6C). We conclude that activation of GPR56 is not mediated by the enzymatic activity of TG2 but requires complex formation between the N-terminal domain of GPR56 with the TG2 C-terminal β-barrel domains. This is supported by TG2 interaction studies carried out with an N-GPR56-Fc fusion protein, or domain deletion mutants thereof, which show that amino acids 1-184 of N-GPR56 (pentraxin and laminin/neurexin/sex hormone-binding globulin-like domain) harbour the TG2 binding site (Supplementary Figure S5). Kinetic studies identified moderately high affinity binding, K_D_ ~1.2 μM, of human TG2 to human N-GPR56 in the presence of Ca^2+^ (but not GTP) (Supplementary Figure S6), which is in line with a recent study investigating the interaction of mouse TG2 with mouse GPR56 (K_D_ ~0.33 μM [42]), and in the range of ligand affinities observed for other adhesion receptors.

To further dissect the domain requirement for TG2-dependent signalling we tested a ΔSTP-GPR56 mutant lacking the proposed TG2 binding site, as well as GPR56 mutants lacking potential C-terminal phosphorylation sites for activity. Figure 6D shows that ΔSTP-GPR56 was not stimulated by TG2 and that this mutant was only partially processed at the GPS site, with C-terminal domain aggregates found in cell lysates by Western blotting (Figure 6E,F). Immunolabeling and confocal analysis revealed that the majority of transfected cells showed only weak or a lack of cell surface ΔSTP-GPR56 staining, indicative of defective trafficking (Figure 5G). In contrast, GPR56 mutants lacking potential serine and threonine cytoplasmic tail phosphorylation sites were abundantly expressed at the cell surface (Figure S3) and also activated by TG2 using AP-AR shedding (Figure 6H).

**Figure 7:**
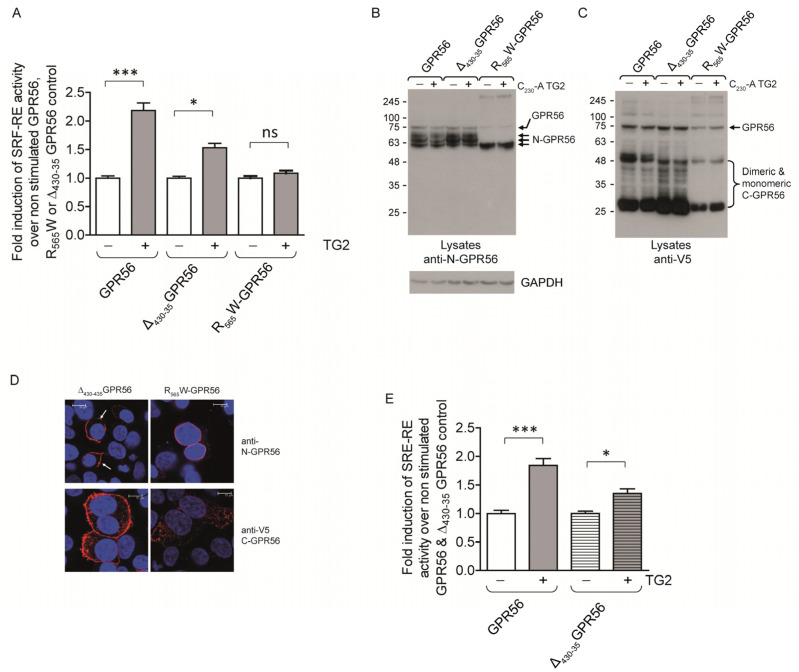
Comparison of the relative signalling activities of wild type GPR56, Δ_430-435_GPR56, and R_565_W-GPR56. (**A**) Comparison of the TG2 signalling response in GPR56, Δ_430-435_GPR56, and R_565_W-GPR56-expressing cells measuring SRF-RE activity, and thus RhoA activation. Δ_430-435_GPR56 is activated, but not R_565_W-GPR56 (*n* = 3). (**B**,**C**) Western blot analysis of GPR56, Δ_430-435_GPR56, and R_565_W-GPR56 using N-GPR56 and V5 epitope antibody in cell lysates, showing correct processing and glycosylation for GPR56 and Δ_430-435_GPR56, but not R_565_W-GPR56, which shows a reduced molecular mass for the N-GPR56 fragment. (**D**) Confocal analysis of Δ_430-435_GPR56 and R_565_W-GPR56 cell surface expression levels using N-GPR56 (top panel) and V5 epitope antibodies (bottom panel), demonstrating significant loss of cell surface localisation for R_565_W-GPR56. Scale bar 10µm. Red GPR56 staining using either anti-N-GPR56 or anti-V5 antibodies. Surface staining for Δ_430-435_GPR56 is indicated using white arrows. Blue nuclear stain. (**E**) Comparison of MAPK activation using SRE-RE luciferase activity in response to TG2 stimulation of wild type GPR56 and Δ_430-435_GPR56 expressing cells (*n* = 3). *p* values below 0.05 (*) were considered significant. *p* values of <0.001 equals (***).

To further evaluate residues in the C-terminal domain of GPR56, we investigated a BFPP disease associated mutant, R_565_W-GPR56. The mutation removes a positive charge in the extracellular loop 2 of GPR56 and constitutes a loss of function mutation. Figure 7A shows lack of Rho activation by TG2 in cells expressing R_565_W-GPR56. Western blot analysis showed alterations in N-GPR56 glycosylation, as indicated by the reduced molecular mass for its N-terminal domain (Figure 7B,C). R_565_W-GPR56 showed a trafficking defect with dramatically reduced cell surface expression levels (Figure 7D), which has been previously confirmed [24].

We also investigated the splice variant Δ_430-435_GPR56, which lacks 5 residues at the beginning of the C-terminal domain in intracellular loop 1, for its ability to be stimulated by TG2. Figure 7A shows a reduced level of activation by TG2 for Δ_430-435_GPR56 using SRF-RE reporter assay compared to full length GPR56. Δ_430-435_GPR56 was highly expressed at the cell surface, thus it is not clear why the Δ_430-435_GPR56 splice variant shows reduced signalling in response to TG2-ligand, which was also seen for SRE-RE reporter activity (Figure 7E). Given we observed reduced reporter activity for both Rho and MAPK activation, it appears that there are true differences in the signalling properties of Δ_430-435_GPR56.

### 2.7. TG2 Is Internalized Selectively in GPR56 Expressing Cells

**Figure 8:**
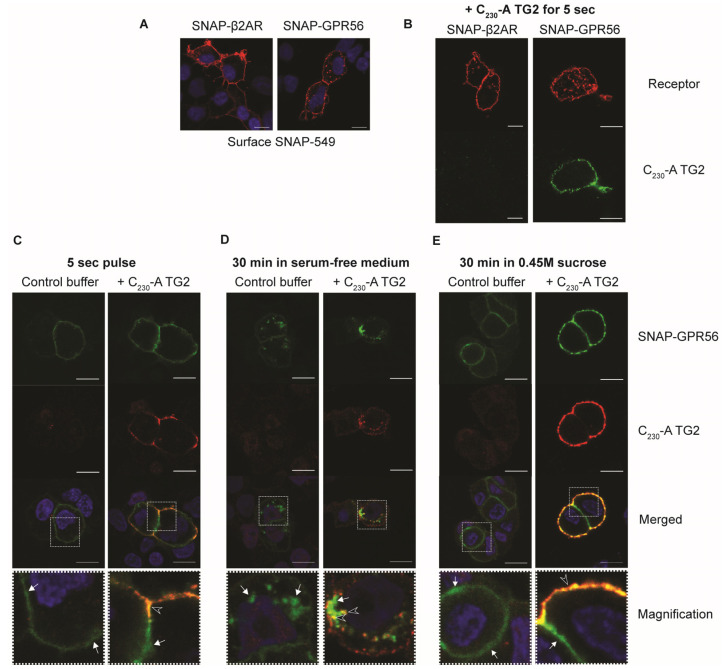
TG2 is internalised rapidly in GPR56-expressing cells, and partially co-localises with N-GPR56 in endocytic vesicles. (**A**) SNAP-β2AR- and SNAP-GPR56-expressing cells were stained with SNAP-surface substrate at 37 °C for 30 min, fixed, and analysed by confocal microscopy. Note the rapid ligand-independent internalisation in SNAP-GPR56 positive cells. Nuclear staining in blue. Red corresponds to SNAP-labelled SNAP-β2AR or SNAP-labelled GPR56 as indicated. (**B**) SNAP-β2AR and SNAP-GPR56 surface-stained cells treated for 5 s with 20 µg/mL C_230_-A TG2. Only SNAP-GPR56-expressing cells bind C_230_-A TG2 (green label corresponds to C_230_-A TG2 and red labels show SNAP-labelled SNAP-β2AR and SNAP-labelled GPR56 respectively). (**C**–**E**) SNAP-GPR56 cells labelled at 4 °C with SNAP surface substrate (green), treated with buffer or C_230_-A TG2 for 5 s (C_230_-A TG2 shown in red). (**C**) and incubated for 30 min at 37 °C in the absence (**D**) or presence of 0.45 M sucrose (**E**). Merged images are shown in the third row. The white square was chosen and further magnified on the confocal microscope (Magnification row). White arrows show SNAP-staining for GPR56. The yellow colour indicates colocalization of GPR56 and TG2 see arrowheads in the Magnification row. Size bar 10 µm.

We then investigated whether C_230_-A TG2 and GPR56 were behaving like a typical ligand receptor pair. SNAP-GPR56 was used to specifically and covalently label cell surface GPR56. Initial experiments compared SNAP-β2AR and SNAP-GPR56 labelled for 30 min at 37 °C, which showed intense surface staining for these receptors. However, some ligand-independent internalization for GPR56 was apparent (Figure 8A). C_230_-A TG2 incubation for 5 s shows specific cell surface staining for TG2 in SNAP-GPR56-expressing cells only, indicating fast and selective binding (Figure 8B). SNAP-surface labelling was then performed on ice to minimize receptor endocytosis during labelling. SNAP-GPR56-labelled cells were treated for 5 s with C_230_-A TG2, washed, and either fixed immediately or incubated in serum-free medium in the presence or absence of sucrose to prevent internalization. Cells were stained with a monoclonal antibody recognizing TG2 and analysed by confocal microscopy for co-localization of ligand and receptor. Figure 8C shows considerable co-localization of C_230_-A TG2 and SNAP-GPR56 following a 5 s pulse treatment, indicating rapid binding between ligand and receptor. Incubation of cells at 37 °C for 30 min showed rapid loss of cell surface SNAP-GPR56-staining in buffer control, as well as in C_230_-A-TG2-treated cells (Figure 8D). There was partial co-localization of C_230_-A TG2, and SNAP-GPR56 in endocytic vesicles at 30 min (Figure 8D, merged magnified image). In contrast, when cells were incubated in the presence of 0.45 M sucrose with C_230_A TG2, SNAP-GPR56, as well as C_230_-A TG2, endocytosis was completely blocked (Figure 8E).

## 3. Discussion

### 3.1. ADAM17 Activation by GPR56 and TG2 Establishes a Ligand Receptor Pair

Although the GPR56–TG2 interaction had previously been shown [11], direct signalling of GPR56 in response to TG2 has been elusive. Our finding that GPR56-dependent AP-AR shedding is enhanced by TG2 demonstrates a signalling response, and this is absent in cells expressing C-GPR56 lacking the N-terminal GPR56 domain that interacts with TG2. ADAM17 inhibition ablated the signalling response, in line with ADAM17 activity being controlled by G_αq_ and G_α12/13_ G-protein signalling [43], as demonstrated for ~100 human GPCRs using TNF-α shedding. Our data show that GPR56 and TG2 are a bona fide ligand/receptor pair for the first time.

GPR56 expression alone causes high ADAM17 activity in the absence of ligand stimulation, which is in line with the literature data showing RhoA activation in response to GPR56 transgene expression [19,20,41]. In agreement with this, GPR56-dependent AP-AR shedding required ROCK activity, in addition to ADAM17 activity. The mechanism for auto-activation is at present unclear, but unrelated to TG2, as it is not expressed in HEK293 cells. Given the high baseline receptor activity and comparably modest ligand-mediated activation, we confirmed the TG2 signalling response using an independent readout. SRF-RE and SRE-RE luciferase reporter assays to monitor Rho kinase and MAPK signalling were used in conjunction with signalling-incompetent N-GPR56 and constitutively active C-GPR56 as controls. They confirm ligand stimulation by TG2 only in cells expressing GPR56, which was also seen for agonistic antibody, which gave comparable activation. As a consequence of GPR56 auto-activation, the apparent level of luciferase stimulation upon ligand binding was 2–3-fold for TG2 or agonistic antibody, and does not compare to classical GPCRs, such as the TSH receptor, which reach >100-fold stimulation levels [44]. However, the other proposed ligand, collagen III [21], was inactive in our assays. The modest increase in activity might be an intrinsic problem that has hampered the study of adhesion GPCRs and may relate to the relative abundance of ligands compared to ligands for classical GPCRs. Additionally, shedding of the N-terminal receptor domain may lead to loss of ligand binding sites [45], and N-GPR56 may potentially associate with the C-terminal 7TM domains of other adhesion GPCRs [46]. These complications explain the increasing evidence that N- and C-terminal adhesion receptor domains convey bimodal activities, as shown for latrophilin-1 [47]. This may also be the case for GPR56, as TG2-binding-deficient ΔSTP-GPR56 was able to activate PKCα, driving VEGF synthesis [48]. This could be causally linked to increased arrestin-β2 association, as seen upon C-GPR56 expression [20] and is consistent with increased levels of C-terminal GPR56 multimers in cells expressing ΔSTP-GPR56.

### 3.2. GPR56 Domain Requirements for Agonist-Mediated ADAM17 Activation

To address whether a functional arrestin platform was required for signalling, we mutated potential G-protein receptor kinase phosphorylation sites in the C-terminus of GPR56. Mutants lacking tail phosphorylation sites were stimulated by TG2 efficiently. Likewise, a cytoplasmic tail deletion mutant was competent to respond to the agonist (Figure S4A). Hence, ADAM17 activation is independent of potential cytoplasmic tail interactions with GPR56. However, the 50 kDa C-terminal receptor fragment was rapidly lost upon TG2 stimulation of wild type GPR56, but not in cells expressing the phosphorylation site mutants, suggesting that these docking sites might be important for receptor uptake and trafficking.

ΔSTP-GPR56 that lacks part of the TG2 interaction site [48] was inert to ligand stimulation, similar to C-GPR56. However, the lack of clear cell surface localization and presence of abundant unprocessed ΔSTP-GPR56 in cell lysates indicates protein-folding or trafficking problems, behaviour reminiscent to that of the well-characterized BFPP mutant R_565_W-GPR56 [15]. In addition to the inherent limitations of the solid phase assay used to localize interaction sites between the receptor and TG2 [11], a previous study questioned whether human, or only murine, TG2 can interact with GPR56 [14]. This led us to further investigate a potential direct interaction. Firstly, an N-GPR56-Fc-fusion protein efficiently bound both wild type human and murine TG2, as well as C_230_A TG2, in solution (Figure S5). A direct interaction between GPR56 and mouse TG2 requiring the C-terminal domain of TG2 was confirmed by Salzman et al., demonstrating binding to the conserved residues on the pentrataxin/laminin–neurexin sex-hormone-binding globulin-like domain of GPR56 [42]. This interaction was interrupted by monobodies. Our data support this finding, but we show additionally that a TG2 mutant lacking both β-barrel domains, while still harbouring enzymatic activity, was unable to trigger a signalling response in GPR56-expressing cells. These data substantiate that the C-terminal domains of TG2 are required for activation of GPR56 and mediate a direct interaction with the N-terminal domain of the receptor. Additionally, the N-terminal domain of GPR56 interacts with heparin, with BFPP mutants R_565_W-GPR56, Y_88_C, or C_91_S displaying higher binding affinity than the wild type protein, and this interaction suppresses GPR56 receptor shedding and promotes cell adhesion and motility [45]. Interestingly, preincubation with heparin diminished the type III collagen interaction with GPR56, but not the TG2 interaction with GPR56.

### 3.3. GPR56 Is Required for Rapid Endocytosis of TG2, Leading to Partial Co-Localisation of TG2 and N-GPR56 in Endocytic Vesicles

Cell surface association of TG2 is generally ascribed to interaction with a fibronectin integrin and syndecan-containing signalling complex [49] that regulates cell-matrix interactions and cell motility [5]. We show that GPR56-expressing cells, but not control cells, rapidly bind TG2, indicating a specific and high-affinity interaction. This is in line with recent observations that monoclonal antibodies blocking the fibronectin binding site do not abolish high affinity binding of TG2 to select tissues [50], as well as a report showing TG2 uptake by GPR56-expressing tumour cells [35]. Consequently, GPR56 may compete for TG2 binding with other cell surface proteins in a cell-type-specific manner, a fact that may have been missed in previous studies.

GPR56-expressing cells internalized TG2 rapidly. Furthermore, TG2 binding did not lead to the release of N-GPR56 into the medium. Therefore, to allow the assessment of co-localization of TG2 with N-GPR56 following uptake, experiments with cell surface labelled SNAP-tagged GPR56 were conducted. SNAP-tagged GPR56 localized to the cell membrane. In the presence of TG2, we confirmed partial co-localisation of TG2 with SNAP-labelled N-GPR56 domain at the cell membrane, and then in endocytic vesicles after 30 min. This behaviour is characteristic for a ligand receptor pair (reviewed in Ref. [51]). Appropriate control experiments demonstrated rapid internalisation of cell surface SNAP-tagged GPR56 in the absence of TG2, indicating that GPR56 has a high internalisation rate due to autoactivation or binding of an alternative ligand [14]. Both TG2-dependent and independent GPR56 uptake was blocked by treatment with 0.45 M sucrose, implicating clathrin-coated pits. Given that the N-GPR56 TG2 interaction was associated with rapid endocytosis of the ligand-receptor complex, internalization may well be required for full receptor activation, as indicated by others using a different approach [20]. A recent study by Olaniru et al. (2021) also showed constitutive internalisation of GPR56 using live super-resolution imaging and SNAP-tagged GPR56, with type III collagen ligand addition, increasing the speed of GPR56 internalization and leading to subsequent cell surface loss of the receptor [52]. Thus, both proposed GPR56-ligands, TG2, and type III collagen promote rapid internalisation of GPR56. This contrasts with previous work, which reported that TG2/laminin or type III collagen addition resulted in N-GPR56 domain release into the medium due to GAIN domain-mediated cleavage [53].

### 3.4. TG2-Dependent Re-Epithelialisation Requires ADAM17-Dependent EGFR Transactivation

While no overt abnormalities in development are evident in TG2 null mice, challenging the mice highlighted substantial deficiencies in tissue repair, including delayed healing of excisional skin defects in different isogenic mouse lines [6,54]. Fibroblasts play a central role in orchestrating the repair response, including re-epithelialisation, angiogenesis, and inflammation. We have previously shown that TG2 is a key factor controlling fibroblast activities in wound healing [5]. TG2 deposition in the ECM and ECM crosslinking occurs rapidly following injury [55], for example, after grafting of burn patients with cultured keratinocyte autografts [56] and is required for the re-establishment of a functional dermo-epidermal cohesion apparatus. Here, we investigated the impact of matrix changes on the re-epithelialisation process using an organotypic culture model based on genetically modified fibroblasts. We show that TG2 loss in the mesenchymally derived ECM impairs the re-epithelialisation response, and, using a series of pharmacological agents, that the role of TG2 in promoting keratinocyte motility is not related to crosslinking activity or enhanced matrix tension, but is mechanistically linked to the activation of ADAM17-dependent EGFR transactivation. This is further supported by the fact that recombinant TG2 can directly and dose-dependently activate ADAM17 in keratinocytes, and that overexpression of a catalytically deficient TG2 C_277_S mutant, which interferes with generation of ECM tension in a collagen lattice contraction assay [5], promotes re-epithelialisation (not shown). It is well established that EGFR transactivation is required in vivo for skin wound healing, as conditional ablation of ADAM17- or EGFR-expression in keratinocytes led to re-epithelialisation defects as well as loss of barrier function in these mice [4]. However, we show for the first time that ADAM17 activity in keratinocytes is regulated by TG2 as a locally acting signalling molecule. Reduced epithelial motility was observed in TG2−/− mice compared to wild type mice following corneal injury, indicating that this mechanism may have a more general role in the regulation of epithelial regeneration [8]. Besides expression in normal epithelia, GPR56 is overexpressed in various ectodermally derived cancers, including SCC and aggressive glioblastoma [25,57]. Acquisition of an aggressive phenotype, as evidenced by enhanced motility or ability to invade, may be explained by increased ADAM17-mediated EGFR transactivation in such tumours.

## 4. Material and Methods

### 4.1. Chemicals and Antibodies

SNAP-tag reagents and plasmids were from New England Biolabs (Ipswich, MA, USA). Luciferase reporter plasmids and reagents were from Promega (Southampton, UK). Oligonucleotides were from MWG Eurofins (Wolverhampton, UK), that sequenced expression constructs and confirmed mutations. R&D systems supplied N-GPR56 (AF4634), EGF (MAB236), HB-EGF (MAB2591), TGF-α (AF-239-NA) antibodies, Invitrogen (now Thermo Fisher Scientific, Waltham, MA, USA) anti-V5 (R-96025), Alexa dye, secondary antibody conjugates and Thermo Scientific anti-TG2 (CUB7402) antibody, Millipore (now Sigma, Burlington, VT, USA) anti-fibronectin (AB2033), NeoMarkers (Freemont, CA, USA) anti-fibrillin-1 (clone 11C1.3, MS-231), and Dako HRP-conjugate to human IgG (P0214). The ADAM inhibitors GW280264x or GI254023x were a kind gift from Dr Augustin Amour, GlaxoSmithKline (Brentford, UK). 

### 4.2. Re-Epithelialisation Model

#### 4.2.1. Production of TG2+ and TG2− ECM

Immortalized human dermal fibroblasts (HCA2) stably expressing various constructs for modulating TG2 expression were characterized previously [5,58]. HCA2 cells were propagated in DMEM supplemented with 10% heat inactivated FCS, 100 µg/mL streptomycin, 100 units/mL penicillin, and 400 µg/mL G418 (Life Technologies, Carlsbad, CA, USA). For matrix production, 1.5 × 10^5^ cells were seeded into each well of a 24-well plate (Falcon 3047; Corning, NY, USA) and grown to confluence. Cell monolayers were washed with PBS and cultured in DMEM containing 2%FCS and 2 mM ascorbate-2-phosphate for 10 days, with medium changed every 2 days. The ECM was washed with PBS and three successive freeze–thaw cycles ensured that HCA2 cells were devitalized. The ECM was treated with 1% sodium deoxycholate/PBS to remove cell debris and washed with PBS. To allow direct comparison of the ECM of different HCA2 cell lines, different cell lines were seeded on a single plate using six replicas.

#### 4.2.2. Preparation of Fluorescently Labelled Keratinocyte Spheroids

N-tert1 keratinocytes [37] were labelled with a PKH26GL red fluorescent cell linker kit (Sigma, Burlington, VT, USA). A total of 1.25 × 10^5^ cells were washed in PBS, re-suspended in 25 µL diluent C, mixed with 50 µL of 100 µM PKH26 solution for 5 min at room temperature, and the reaction stopped by adding 50 µL FCS. Cells were washed and uniform spheroids generated by incubating keratinocytes in a hydrophobic 96-well plate (2500/well; Greiner Bio-one, Kremsmünster, Austria) in FAD medium supplemented with 0.39 g/mL methylcellulose for 20 h.

#### 4.2.3. Migration Studies on TG2+ and TG2− ECM Using Fluorescently Labelled N-Tert1 Keratinocytes Spheroids by Time Lapse Microscopy

Spheroids were washed and transferred onto devitalized fibroblast TG2+ or TG2− ECM by pipetting 50 µL medium containing a single spheroid, where medium was supplemented with MMP or ADAM inhibitors, receptor kinase inhibitors, and relevant carrier controls. Six replicas were performed for each condition per experiment in FAD medium containing 1% FCS lacking specified supplements, or in serum-free keratinocyte medium containing bovine pituitary extract (Invitrogen).

Timelapse microscopy was carried out using a motorized Zeiss Axiovert inverted microscope controlled by Openlab^TM^ software (Version 4.1.2, Improvision; now PerkinElmer, Shelton, CT, USA) [5]. Fluorescence and DIC images were acquired hourly to follow radial N-tert1 keratinocyte migration. Greyscale images were imported into CTAn software (Version 1.5.1.5 and version 1.10.0.1; Skyscan, now Bruker, Billerica, MA, USA), a defined threshold applied for conversion into binary images, and the ‘shrinkwrap’ algorithm used for calculation of surface area covered by epithelium (Supplement Figure S1A). Data are expressed as average radial distance of cell migration front over time from the original spheroid boundary.

#### 4.2.4. Expression Constructs and Characterization of TG2, C_230_-A TG2, C_277_-S TG2, and N-Terminal TG2

The human C_230_-A TG2 mutant was prepared by overlap extension mutagenesis using primers described in [32] and cloned into a rhamnose-inducible expression plasmid for human TG2 [59]. An N-TG2 expression plasmid was created using PCR, and a stop codon inserted at position 471. All TG2 proteins were expressed in *E. coli* BL21(DE3pLysS) (Promega) and purified to homogeneity [59]. Enzymatic activity was measured as monodansylcadaverine incorporation into N,N-dimethylcasein and cleavage of the internally quenched fluorescent substrate Abz-APE(γ-cad-Dnp) QEA (Zedira, Darmstadt, Germany) [60]. TG2 was incubated with a 10-fold molar excess of GTPγS for 30 min at 4 °C to form TG2-GTPγS, and free nucleotides were removed using a PD10 column (GE-Healthcare, Chicago, IL, USA). Ca^2+^-activated TG2 was prepared by adding 1 mM CaCl_2_ prior to cell stimulation.

##### Expression Constructs for N-GPR56-Fc Fusion Proteins

The pSecTag2 expression vector version B (Invitrogen) was modified through insertion of the human IgG_1_ heavy chain coding sequence (amino acids 3-236) into the BamHI and EcoRI restriction sites of the multiple cloning site. The coding sequences for N-GPR56, as detailed in Figure S5A, were amplified by PCR (Supplementary Table S1) and cloned into the NheI and BamHI restriction sites to yield an Fc-fusion protein lacking any spacer sequence but retaining the appropriate cysteine residues for dimerization. The sequence of the expression constructs was verified by Sanger dideoxy DNA sequencing (Eurofins MWG). The graphical abstract and Supplementary Figure S5 were prepared using Biorender.

##### Expression of N-GPR56-Fc Fusion Proteins in CHO Cells

CHO cells were seeded at 1.5 × 10^5^ cells in 6-well plates and grown to 70% confluence. Cells were transfected with 2.0 µg N-GPR56-Fc-expression plasmid using LT1-CHO (TransIT-CHO transfection kit MIR 2170, MirusBio, Madison, WI, USA) at a ratio of 3 µL lipids per µg DNA and according to the manufacturer’s instructions. Briefly, DNA-lipid complexes were prepared in serum-free Ham’s F12 media (Invitrogen, 21765-037) by incubation for 20 min and supplemented with Mojo reagent and serum free medium to yield a total of 1 mL. Cells were washed twice with serum-free medium and supplemented with 1 mL Ham’s F12 media containing 10% heat-inactivated ultra low-IgG FBS (Life Technologies, Carlsbad, CA, USA) before dropwise addition of DNA-lipid complexes to the cell monolayer. Cells were incubated for 36 to 48 h at 37 °C/5% CO_2_. Conditioned medium (2 mL) was removed, 20 µL of 1 M Tris/HCl, pH7.4 added to buffer the pH, centrifuged for 10 min at room temperature at 1500× *g* to remove cellular debris, snap frozen, and kept at −20 °C until further use.

##### Purification of N-GPR56-Fc Fusion Proteins

Thawed conditioned media (1.0 mL) was centrifuged at 10,000× *g* for 5 min at 4 °C to remove any precipitates prior to incubation with washed 40 µL of 75% slurry of protein G-Sepharose beads (GE Healthcare, 17-0618-01; wash buffer 20 mM Tris/HCl, pH 7.4, 150 mM NaCl) overnight at 4 °C on a rotating shaker. Beads were collected by centrifugation (1500× *g* for 1 min at 4 °C) and then washed a minimum of 3 times using 500 µL wash buffer. N-GPR56-Fc fusion proteins were eluted from the protein G-beads with 50 µL 0.1 M glycine/HCl, pH 2.5, for 5 min at 4 °C while shaking, followed by centrifugation at 5000× *g* for 1 min at 4 °C. The supernatant was collected and immediately neutralized with 2 µL 2 M Tris/HCl, pH 9.0. For Western blotting, the proteins were concentrated through lyophilization, and finally dissolved in Laemmli sample buffer containing 2% 2-mercaptoethanol. Proteins were separated in Novex 4–20% Tris/glycine SDS-PAGE gels, and immunoblotting conducted, as described in the relevant section, using an anti-human IgG-Fc/HRP conjugate for detection.

##### TG2 Pull Down Assay

Protein G-beads harbouring N-GPR56-Fc fusion proteins were prepared, as described above. Following washing, beads were incubated with human TG2 (50 µg/mL final concentration) in a volume of 50μL overnight at 4 °C on a rotary shaker. Beads were collected by centrifugation (1500× *g* for 1 min at 4 °C) and then washed a minimum of 5 times using 100 µL wash buffer. Finally, the N-GPR56-Fc-fusion—TG2 complexes were eluted from protein G by addition of 100 µL of 0.1 M glycine/HCl, pH2.5, by incubation for 15 min on ice, and analysed by Western blotting, as described above.

##### Surface Plasmon Resonance Analysis

For kinetic studies, C_277_S-TG2 was incubated with 10 mM DTT for 10 min at room temperature before being re-buffered into Biacore HBS running buffer (10 mM Hepes, pH 7.4, 0.15 M NaCl, 3 mM EDTA, 0.005% (*v*/*v*) surfactant P20) using PD SpinTrap 25 columns (GE Healthcare, Chicago, IL, USA). Binding analysis was performed using a BIAcore T200™ equipped with a protein A sensor chip (series S, 29127555). Binding analysis was performed 4 times in independent experiments. Approximately 270–320 response units (RU) of 1-382 N-GPR56-Fc fusion protein or fusion protein containing conditioned media (diluted 1:10 in HBS buffer) was attached to the protein A sensor chip at 10 μL/min to ensure uniform distribution on the surface. Buffer or conditioned media from untransfected cells was used for control binding studies. Combined with the small amount of N-GPR56-Fc-fusion protein bound to the chip surface, this reduced the likelihood of off-rate-limiting mass transfer effects. All measurements were performed at 25 °C in HBS buffer (GE Healthcare) at a flow rate of 30 µL/min. Where indicated, 3 mM GTP or 20 mM Ca^2+^ was included. For equilibrium analysis, 8 serial dilutions were prepared for each analyte, injected over the chip surface using kinetic injections. Results were analysed using BIAevaluation 3.1^TM^ software and GraphPad PRISM version 6. The equilibrium-binding constant (K_D_) values were derived from a nonlinear curve fit (y = (P_1_x)/(P_2_ + x)).

#### 4.2.5. Keratinocyte Proliferation

N-tert1 cells (5 × 10^3^/well) were seeded into 24-well plates, and after 6 h were switched to 1% FCS FAD medium lacking EGF and stimulated with TG2 variants, as indicated. At 24 or 72 h, cells were incubated with 1 mg/mL MTT for 4 h, and subsequently formazan was quantified at 570 nm. Cell numbers were derived from a standard curve, and results were expressed as increase in cell number over 48 h relative to control.

#### 4.2.6. GPR56-Expression Analysis

Primary human keratinocytes were cultured and treated as before [61]. mRNA was prepared using FastTrack Kit (Invitrogen) and converted to cDNA with SuperScriptII. A GPR56 fragment was amplified with: 5′CATGTGCTGACACTGCTGGGC3′ and 5′CTGGCGCTGTCTGAGTTGCTC3′. Relative expression levels were determined by quantitative PCR using SybrGreen core reagents (Applied Biosystems, Waltham, MA, USA).

#### 4.2.7. GPR56-Expression Constructs

GPR56 expression constructs were generated using PCR reactions with primers, detailed in Supplementary Table S1, using either Herculase (Stratagene, San Diego, CA, USA) or Fusion DNA polymerase (Thermo Scientific). Complex mutations were introduced by overlap extension mutagenesis [62,63]. For the tail S-A GPR56 mutant, an intermediate expression construct was generated, designated S_687/689_A GPR56. The S-A GPR56 mutant was used to generate S/T-A GPR56. GPR56-expression plasmids were cloned into pcDNA4/V5/His plasmid in frame with a C-terminal V5/His tag, with exception of N-GPR56, which carries a C-terminal Flag/His tag. For details of the domain boundaries and residues mutated at the amino acid level, see Supplementary Figure S2D. Supplementary Figure S3 shows a detailed analysis of cell surface localisation of GPR56 mutants.

#### 4.2.8. GPR56-Dependent Activation of ADAM17

HEK293 cells were from Invitrogen, and cells were seeded into 24 well plates at 1 × 10^5^ cells/well. The next day, 2 μg wild type or mutant GPR56-expression plasmids were mixed with 1 μg AP-AR-expression plasmid using 9 μL Fugene6 (E2691, Promega, Madison, WI, USA) and used to transfect four wells of a 24-well plate. Two days post transfection, the medium was removed, and cell monolayers were washed with serum-free advanced DMEM and serum starved for 1 h. GPR56 activation experiments were performed by incubating with 20 μg/mL C_230_-A TG2, TG2, N-TG2, 84 nM type III collagen or 5 μg/mL N-terminal GPR56 antibody (1 h). For inhibition studies with ADAM inhibitors, we treated cells with 1 µM inhibitors or DMSO control during serum starvation (1 h) and during the 1 h ligand stimulation, as indicated. Medium was cleared by centrifugation and AP-activity assayed, as described previously [62]. Each experiment was performed with 4 replicas per condition. 4-NPP hydrolysis rates were measured over time to ensure that the reaction was linear and that substrate depletion did not occur. The hydrolysis rates from individual experiments were then normalized by setting the values for the GPR56 buffer/DMSO control to one (Figure 3C,D). Data are the mean +/−s.e.m. from 3–5 independent experiments, as detailed in the figure legends.

#### 4.2.9. Luciferase Reporter Assays

Luciferase reporter assays were performed in 96-well plates using a seeding density of 1 × 10^4^ cells per well. The next day, cells were transfected with 50 ng/well SRF-RE or SRE-RE reporter plasmid and 150 ng/well GPR56 expression plasmids using Fugene 6. Cells were stimulated with appropriate ligands or controls two days later in serum-free conditions (6 h). Cells were lysed in 50 µL passive lysis buffer. A amount of 10 µL lysis solution was assayed for luciferase activity using a BMG Labtech Fluostar Optima (BMG, Labtech, Ortenberg, Germany) plate reader. A total of 12 experimental replicas per condition were used, and data were normalised to buffer control treated N- or GPR56 transfections. In total, 3–4 independent experiments were analysed.

#### 4.2.10. SDS-Page and Western Blotting

Cell lysates (50 μg protein) were incubated in a reducing sample buffer for 10 min at room temperature to avoid induction of aggregation by boiling [14]. Samples were separated using 10% SDS-PAGE and transferred at 15 V for 16 h onto PVDF (Millipore) membranes. Membranes were blocked for 1 h with 5% skimmed milk in TBST and incubated with anti-V5 or anti-N-GPR56 antibodies at 1:5000 or 1:1000 dilution in 5% skimmed milk/TBST at 4 °C (14 h). Primary antibodies were detected with appropriate secondary antibodies conjugated to HRP using ECL.

#### 4.2.11. Immuno-Localisation of GPR56 in Transiently Transfected Cells

HEK293 cells were plated onto poly-l-lysine coated coverslips and transfected with GPR56 plasmids. Immunostaining of GPR56 for the N-GPR56 domain was performed on cells fixed with 4% paraformaldehyde/PBS for 5 min. Cells were washed with PBS and blocked for 30 min in 1% BSA/PBS prior to incubation with primary sheep anti-N-GPR56 antibody (1 μg/mL, 2 h). Alexa Fluor 568-conjugated anti-sheep antibody (4 μg/mL) in the blocking buffer was used to visualize the primary antibody. The C-terminal GPR56 domain was detected via the V5 epitope-tag using the same method, but a 10 min permeabilization step with 0.5% saponin/PBS was included prior to incubation with anti V5-antibody (1 µg/mL). Bound V5 antibody was detected with a secondary Alexa Fluor 594-conjugated anti-mouse antibody (4 μg/mL). Images were acquired by confocal microscopy with a 63× objective at 543 nm excitation.

#### 4.2.12. Internalisation of TG2 and SNAP-GPR56

The SNAPf-tag coding sequence was amplified using pSNAPf-ADRβ2 control plasmid with primers: 5′ATAAGATCTAGCATGGACAAAGACTGCGAAATGAAG3′ and 5′ATATCTAGACTCGAGGGATCCTGGCGCGCCTATACCTGC3′. The PCR product was cloned into a modified pcDNA4/His encoding an N-terminal IgG signal sequence. GPR56 cDNA was then cloned in-frame with the IgG signal N-terminal SNAPf-tag in the pcDNA4/V5/His plasmid. SNAP-GPR56 cDNA was transfected, and cells covalently labelled with SNAP-surface Alexa Fluor 647-substrate (1 µM), at 4 °C for 15 min (48 h post-transfection). Cells were washed with serum-free advanced 0.5% BSA/DMEM prior to TG2 treatment (20 µg/mL) for 5 s. TG2 containing medium was removed, replaced with serum-free medium with or without 0.45 M sucrose. After 30 min cells were fixed with 4% paraformaldehyde/PBS, washed, permeabilised with 0.5% saponin and then washed and blocked with 1% BSA for 30 min. Cells were stained for TG2 using the CUB7402 and Alexa Fluor 594-conjugated anti-mouse antibody. Coverslips were mounted onto slides using 5 μL vecta shield containing DAPI. Images were acquired by confocal microscopy with a 63× objective using excitation at 543 and 633 nm, respectively. Three independent experiments were performed. 

#### 4.2.13. Statistical Analysis

Statistical analysis was performed using One-way Anova with a Tukey post-test (Graph-Pad Prism 5 or 6) unless otherwise indicated. *p* values below 0.05 (*) were considered significant. *p* values of <0.01 equals (**), *p* values of <0.001 equals (***), and *p* values of <0.0001 equals (****), as indicated in the figures.

## 5. Conclusions

Our data show that GPR56 and TG2 are a bona fide receptor ligand pair, and their interaction mediates activation of the disintegrin and metalloproteinase ADAM17. The interaction is mediated by the C-terminal β-barrel domains of the Ca^2+^-enabled ‘open’ conformation of TG2 and the N-terminal pentrataxin/laminin–neurexin sex-hormone-binding globulin-like domain of GPR56, and subsequently results in rapid internalization of TG2 into endocytic vesicles. ADAM17 activation, in turn, drives the release of EGFR-receptor ligands from the cell membrane, and thereby results in the transactivation of EGFR signalling. In keratinocytes, EGFR signalling promotes cell proliferation and motility, and, hence, ECM-associated mesenchymally derived TG2 promotes the re-epithelialisation responses in keratinocytes via this mechanistic pathway, as demonstrated here in an organotypic in vitro model.

## Data Availability

Data are contained within the article or Supplementary Materials.

## Supplementary Materials

The following supporting information can be downloaded at: https://www.mdpi.com/article/10.3390/ijms25042329/s1. References [61,64] are cited in the Supplementary Materials.
