# Supplementary material for: Mesenchymal Transglutaminase 2 Activates Epithelial ADAM17: Link to G-Protein-Coupled Receptor 56 (ADGRG1) Signalling
Source: Int J Mol Sci. 2024 Feb 16;25(4):2329. doi: 10.3390/ijms25042329 (PMC10889368; doi:10.3390/ijms25042329)
Supplement: Supplementary file 1 [file ijms-25-02329-s001.zip › ijms-2846564-supplementary.pdf]

Mesenchymal transglutaminase 2 activates epithelial ADAM17: link to G-protein coupled receptor 56 signalling

Lea Bauer[*], Jessica Edwards[*,‖], Andreas Heil[*,‖], Sharon Dewitt[*], Heike Biebermann[ǂ], Daniel Aeschlimann[*,‡] and Vera Knäuper[*, ‡]

[*]Cardiff University, Dental School, Cardiff, CF14 4XY, United Kingdom
[ǂ]Charité, Virchow-Klinikum, Institute for Experimental and Pediatric Endocrinology, Augustenburger Platz1, 13353 Berlin, Germany.

[‖,‡] These authors contributed equally to this paper.

<u>Supplemental Table 1:</u>
PCR primers used for expression constructs and Q-PCR experiments.

<u>Supplemental Figure S1:</u> Characterization of keratinocyte spheroid migration on TG2+ or TG2- ECM.

(A) Quantitative analysis of epithelial expansion. To objectively quantify epithelial migration over time, original fluorescence images (a) were converted into binary images using a set threshold, and the boundary of epithelial cells migrating from the spheroid determined using a rolling ball algorithm (b). The area covered by epithelial cells was integrated and the distance of average epithelial expansion (Δr) calculated from a circle of equal area (solid line) with the radius of the spheroid at time zero (dotted line) subtracted (c).

(B) Analysis of ECM deposition under different conditions by fibroblasts expressing TG2 (TG2+ECM) or deficient in TG2 (TG2-ECM). The respective HCA2 fibroblast lines were grown to hyperconfluency and then switched onto DMEM supplemented with ascorbate-2-phosphate and various concentrations of FCS as indicated to induce ECM deposition for 10 days. Fibroblast-containing ECM was fixed in 4% paraformaldehyde/PBS and stained with anti-fibronectin or anti-fibrillin-1 antibodies, followed by fluorescently-labeled secondary antibodies. Both, TG2+ and TG2- fibroblasts assemble ECM in the presence of greater than 1% serum. Note, fibrillin-1 fibril deposition does not occur in conventional monolayer culture (Kielty et al., 1994).

(C) Role of metalloproteinases in ECM-induced enhanced keratinocyte spheroid expansion. Time course of keratinocyte migration out of spheroids on TG2+ ECM, showing inhibition by 50μM GM6001 or 10μM TAPI-1, when compared to DMSO carrier control in serum-free keratinocyte medium. Data are average ± s.e.m (n=5).

Supplemental Figure S2: GPR56 is found in human keratinocytes but not dermal fibroblasts.

(A) PCR analysis of GPR56 expression using mRNA isolated from HCA2 fibroblasts (F) and N-tert 1 immortalized keratinocytes (NtK).

(B) Quantitative PCR analysis of GPR56 expression in primary and NtK keratinocytes (±SD, n=2 with duplicates). To analyze GPR56 expression in relation to cell differentiation, normal foreskin keratinocytes were grown post-confluence in the presence of 0.5 nM EGF or KGF, factors known to attenuate differentiation (Aeschlimann et al., 1998).

(C) Western blot analysis of NtK cell lysates showing the N-GPR56 fragment detected with N-GPR56 antibody.

(D) Schematic representation of GPR56 expression constructs used in this study.

Supplemental Figure S3: Cell surface localisation of GPR56 mutants analysed by confocal microscopy.

The expression constructs are indicated above each panel and the antibodies are shown on the left.

(A) The top panels in each row shows transiently transfected cells that were stained with polyclonal anti-N-GPR56 antibody (red) for the presence of the N-terminal domain of GPR56. Arrows indicate cell surface staining detected with anti-N-GPR56 antibody in non-permeabilising conditions. The lower panels in each row are transiently transfected cells, stained with monoclonal anti-V5 antibody (red) for the presence of C-terminal GPR56 using permeabilising conditions.

(B) The top right hand panel shows cells, transiently expressing N-GPR56 (Flag-tagged) stained with the polyclonal anti-N-GPR56 antibody (red) for the presence of the N-terminal domain of GPR56. Arrows indicate cell surface staining detected with anti N-GPR56 antibody under non-permeabilising conditions (red). The bottom right hand panel shows cells transiently expressing N-GPR56 (Flag-tagged) stained with anti-Flag monoclonal antibody (red) using permeabilising conditions.

Supplemental Figure S4:

(A) TG2 dependent AP-AR shedding is independent of the cytoplasmic tail of GPR56 (n=5); for experimental details see Figure 4.

(B) A combination of inhibitory EGF, TNF-α and HB-EGF antibodies suppressed TG2 dependent proliferation in N-tert1 immortalized keratinocytes, whereas inclusion of EGFR ligand antibodies (10μg/ml) individually yielded partial suppression.

Supplemental Figure S5:

Analysis of TG2 binding to N-GPR56-Fc fusion proteins. **(A)**, Schematic representation of N-GPR56-Fc fusion protein expression constructs; **(B)**, Western blot analysis with anti-human IgG antibodies of media from transiently transfected CHO cells expressing the constructs shown in A. Proteins were separated in 4-20% SDS PAGE gels under reducing conditions. M_r markers are indicated on the left. **(C)** Schematic representation of TG2 pulldown experiments using N-GPR56-Fc fusion protein and protein G beads. **(D)**, Western blot analysis of bead wash steps and acid elution step. Staining for human IgG, detecting 1-382 N-GPR56-Fc fusion protein in elution step only, is shown on the left blot. Detection of TG2 with CUB7402 antibody (right blot) shows progressively diminished amounts of TG2 in wash steps but also co-elution of some TG2 with GPR56-Fc fusion protein in elution step. **(E)** Pulldown experiment using all four N-GPR56-Fc fusion proteins as seen in (**A**), whereby only the final wash step and acid elution step are included in the Western blot analysis**.** Staining for human IgG is shown in left blot, detection of bound TG2 revealed in acid elution step following staining with anti-TG2 antibody (right blot).

Supplemental Figure S6:

Analysis of kinetics for the TG2 - GPR56 interaction conducted on Biacore instrument using a protein A sensorchip for immobilization of the Fc fusion protein (1-382 N-GPR56-Fc). As we observed a Ca^{2+}-dependent interaction in a previous experiments (Fig 2A), these experiments were conducted with the active site mutant, C277S-TG2, to avoid simultaneous cross-linking from occurring which would interfere with the analysis. **(A)** A representative sensogram is given ('TG2' denotes the time of analyte injection) for the analysis of C277S-TG2 GTP complex (3mM GTP) as well as the C277S-TG2 in the presence of Ca^{2+} (20mM), showing that almost no binding to immobilized 1-382 N-GPR56-Fc occurs in the absence of Ca^{2+}. This suggests that only the extended 'open' conformation of TG2 adopted in the presence of Ca^{2+} binds effectively to GPR56. **(B)** To further characterise the interaction, analyte concentration-dependent interaction (in the presence of Ca^{2+}) was measured (insert), and is shown for ligand immobilization carried out with either purified 1-382 N-GPR56-Fc fusion protein (GPR56Fc purified) or conditioned medium of CHO cells expressing the respective Fc fusion protein (GPR56Fc media). Equilibrium binding constants (K_D) were derived using nonlinear curve fitting as described in the Methods section. Figure S6 was constructed using Biorender.

<u>Supplemental Table 1:</u>

Primers used for cDNA expression constructs and Q-PCR experiments:

C_{230}-A TG2 +	$^{5'}$GGCATGGTCAACGCCAACGATGACCAGGGTGTGCTGCTGGGA$^{3'}$
C_{230}-A TG2 -	$^{5'}$CCTGGTCATCGTTGGCGTTGACCATGCCACTCACCACCCGGC$^{3'}$
N-TG2 -	$^{5'}$GTCAAGCTTACCCTGTCTCCTCCTTCTCGGC$^{3'}$
GPR56 +	$^{5'}$AAAAGATCTAAAATGACTCCCCAGTCGCTGCTGCAGA$^{-3'}$
GPR56 -	$^{5'}$AATCTAGAGATGCGGCTGGACGAGGTGCTGCCCGAGCT-$^{3'}$
R_{565}W-GPR56 +	$^{5'}$TACCCTTCCATGTGCTGGATCTGGGACTCCCTGGTC$^{3'}$
R_{565}W-GPR56 -	$^{5'}$GACCAGGGAGTCCCAGATCCAGCACATGGAAGGGTA$^{3'}$
N-GPR56 -	$^{5'}$AACTCGAGGTAGGTCAAGTGGTTGCAGAAGCAGGAT$^{3'}$
C-GPR56 +	$^{5'}$ATAGGTAGGATGGGCCTGCAATGTGTGTTCTTCTGGGTTGAAGACCCC$^{3'}$
$S_{687/689}$A GPR56 -	$^{5'}$AAATCTAGAGATGCGGCTGGAAGCGGTGGCGCCCGAGCTGATGGG$^{3'}$
S-A GPR56 -	$^{5'}$AAATCTAGAGATGCGGGCGGCAGCGGTGGCGCCAGCACCGATGGGGAGCCTGGC$^{3'}$
S/T-A GPR56 -	$^{5'}$AAATCTAGAGATGCGGGCGGCAGCGGCGGCGCCAGCACCGATGGGGAG$^{3'}$
Δ-STP-GPR56 +	$^{5'}$GAGGTCCCTTTTGAGCTCCTTGCCATAGAGAAGATG$^{3'}$
Δ-STP-GPR56 -	$^{5'}$CATCTTCTCTATGGCAAGGAGCTCAAAGGGACCTC$^{3'}$
$\Delta_{430-435}$GPR56 +	$^{5'}$TACCTCTGCTCCAGGAGGAAACCTCGGGACTACACCATCAAGGTG$^{3'}$
$\Delta_{430-435}$GPR56 -	$^{5'}$GTCCCGAGGTTTCCTCCTGGAGCAGAGGTAGGCGGCAATGGTGAC$^{3'}$
GPR56 Q-PCR +	$^{5'}$CATGTGCTGACACTGCTGGGC$^{3'}$
GPR56 Q-PCR -	$^{5'}$CTGGCGCTGTCTGAGTTGCTC$^{3'}$
N-GPR56 Fc +	$^{5'}$TAGCTAGCCACCATGACTCCCCAGTCGCTGCTGC$^{3'}$
1-184 N-GPR56 Fc -	$^{5'}$ATGGATCCCTGGAGGTCCCTTTTGAGCTCGCAC$^{3'}$
1-241 N-GPR56 Fc -	$^{5'}$TAGGATCCCTGGAGCTTCCACACCGTGGCGTTG$^{3'}$
1-345 N-GPR56 Fc -	$^{5'}$ATGGATCCTTGCAGAGTCACATTCTTCGGCTG$^{3'}$
1-382 N-GPR56 Fc -	$^{5'}$ATGGATCCCAAGTGGTTGCAGAAGCAGGATG$^{3'}$

Supplemental Figure S1

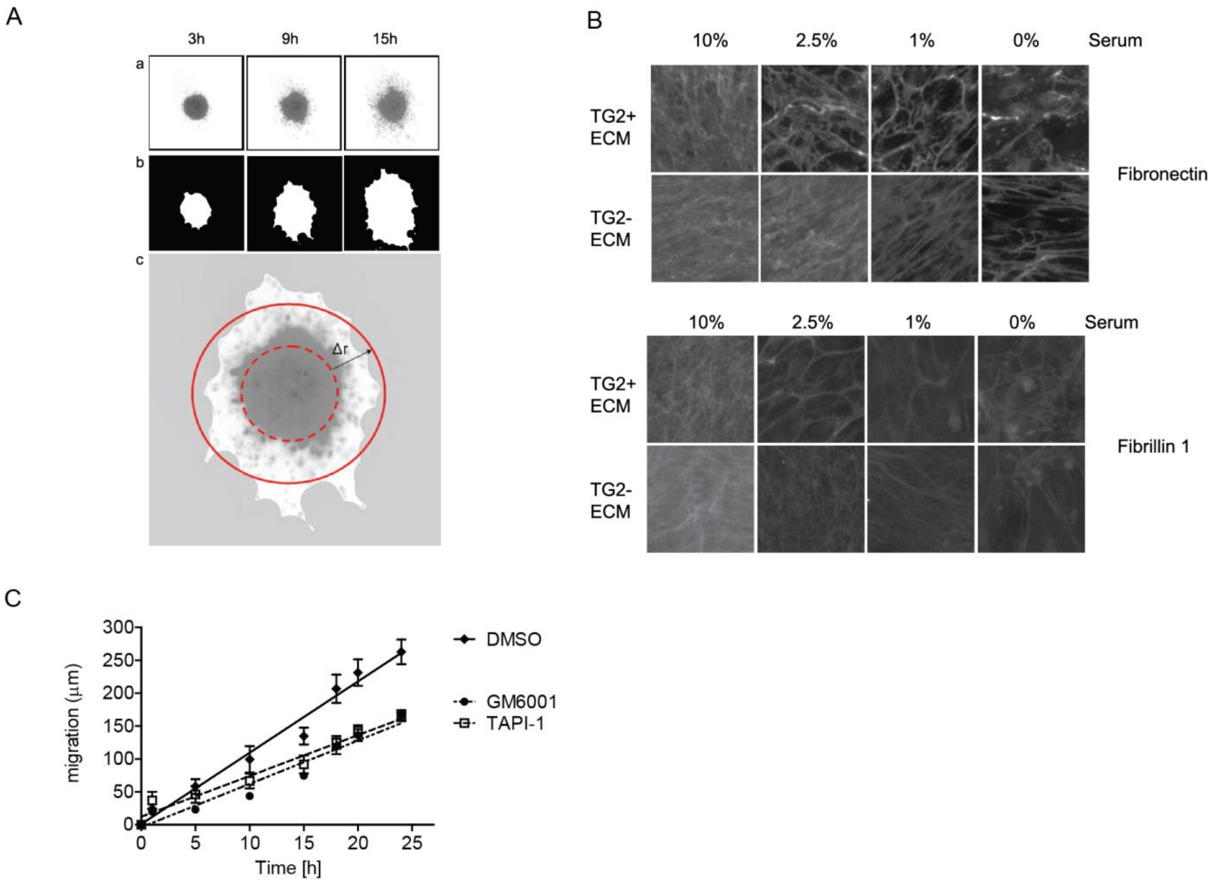

Supplemental Figure S2

Supplemental Figure: S2 A-D

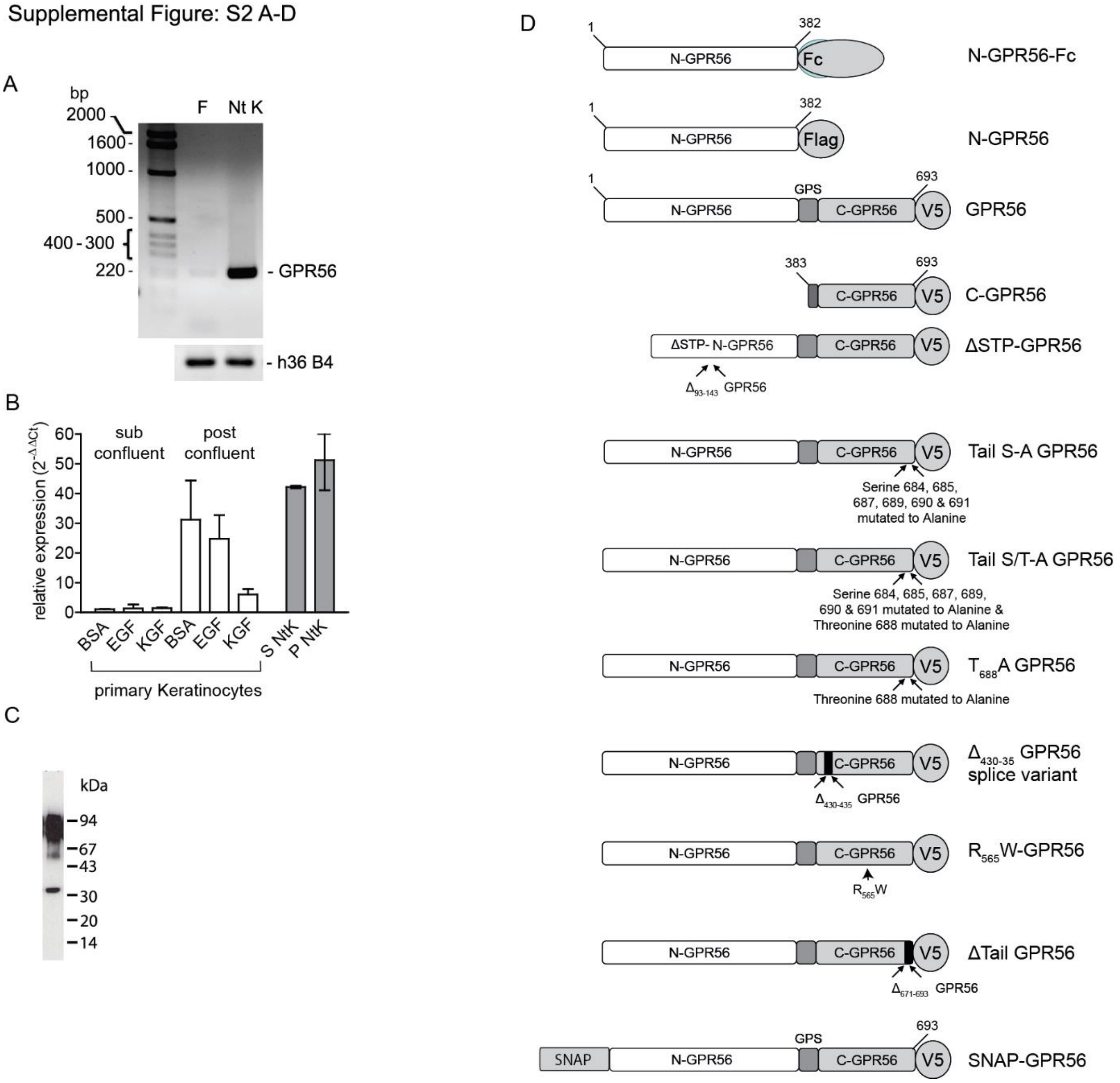

Supplemental Figure S3

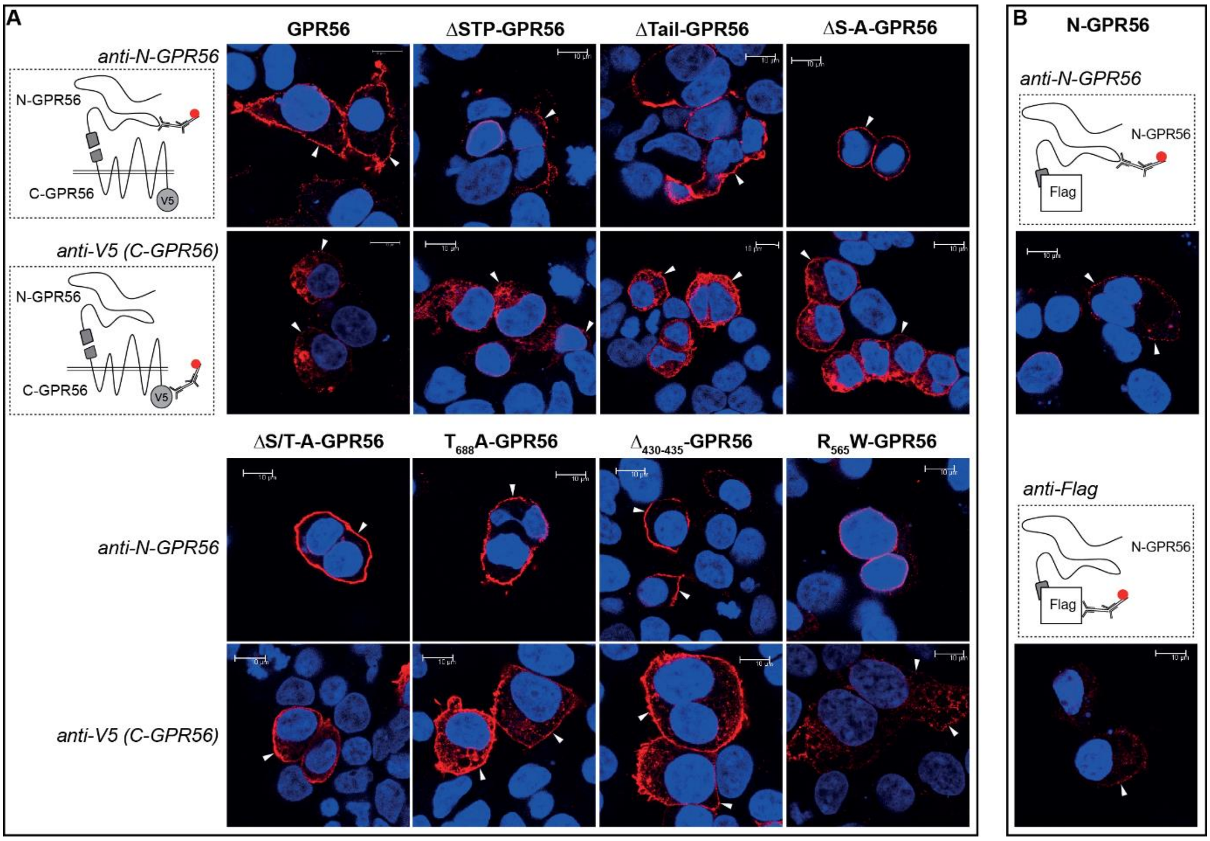

Supplemental Figure S4

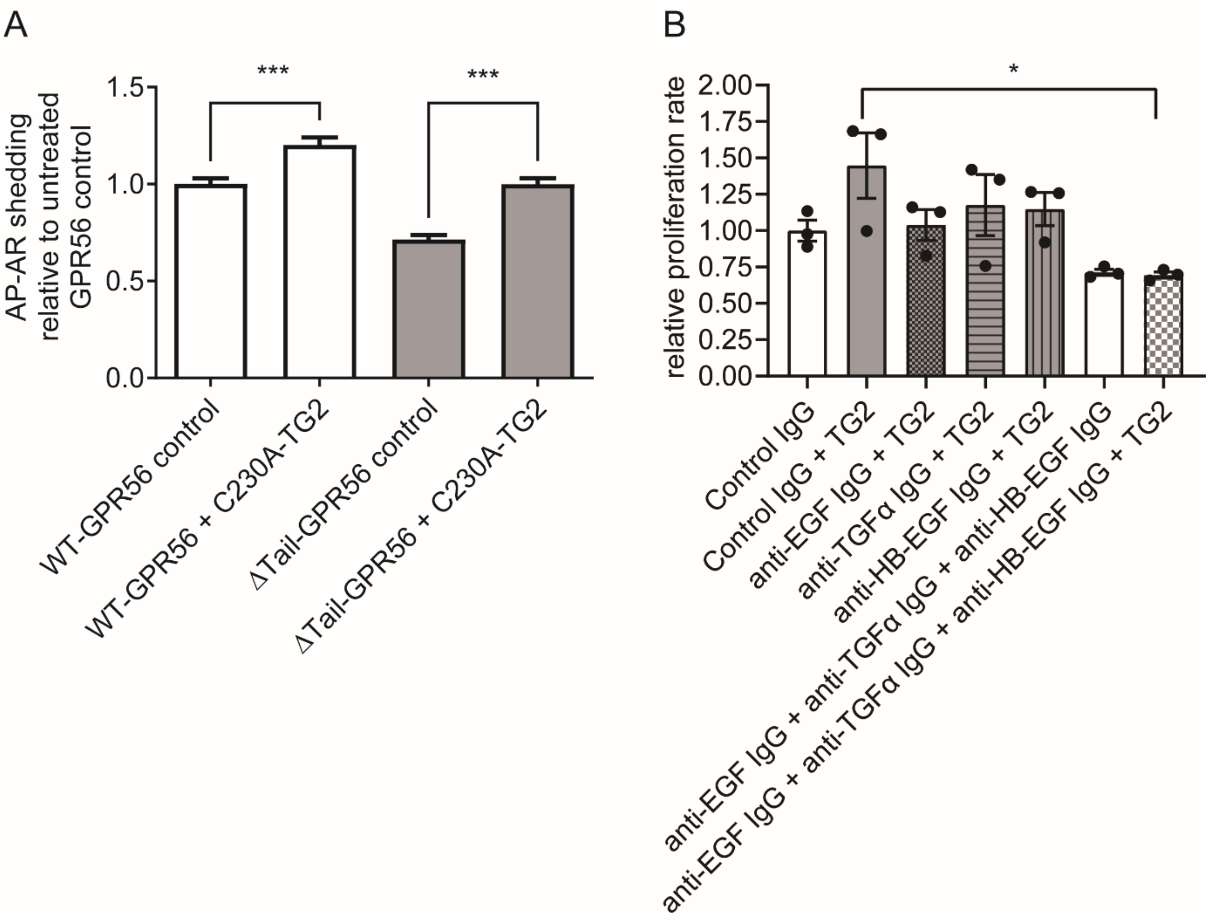

Supplemental Figure S5

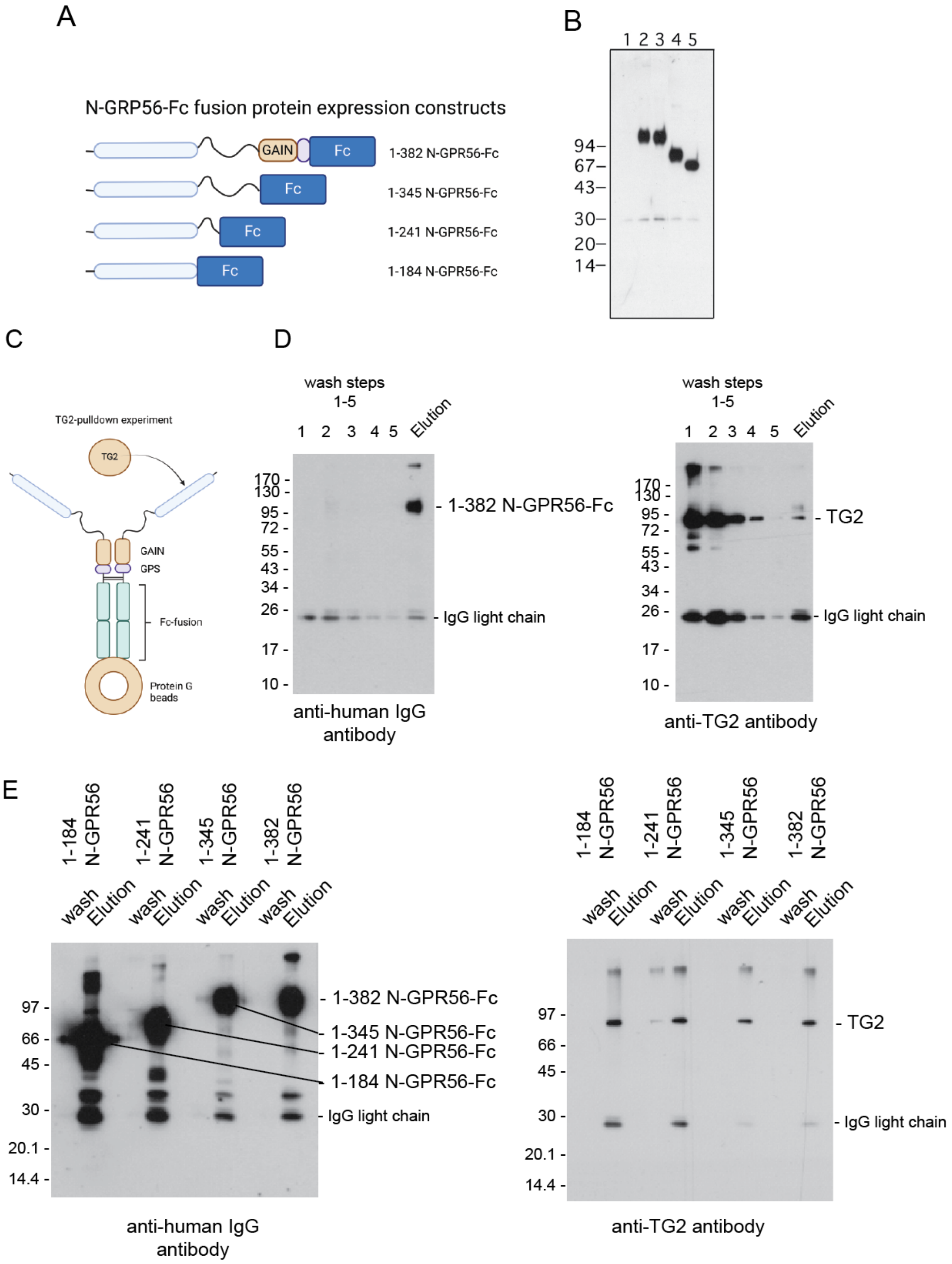

Supplemental Figure S6

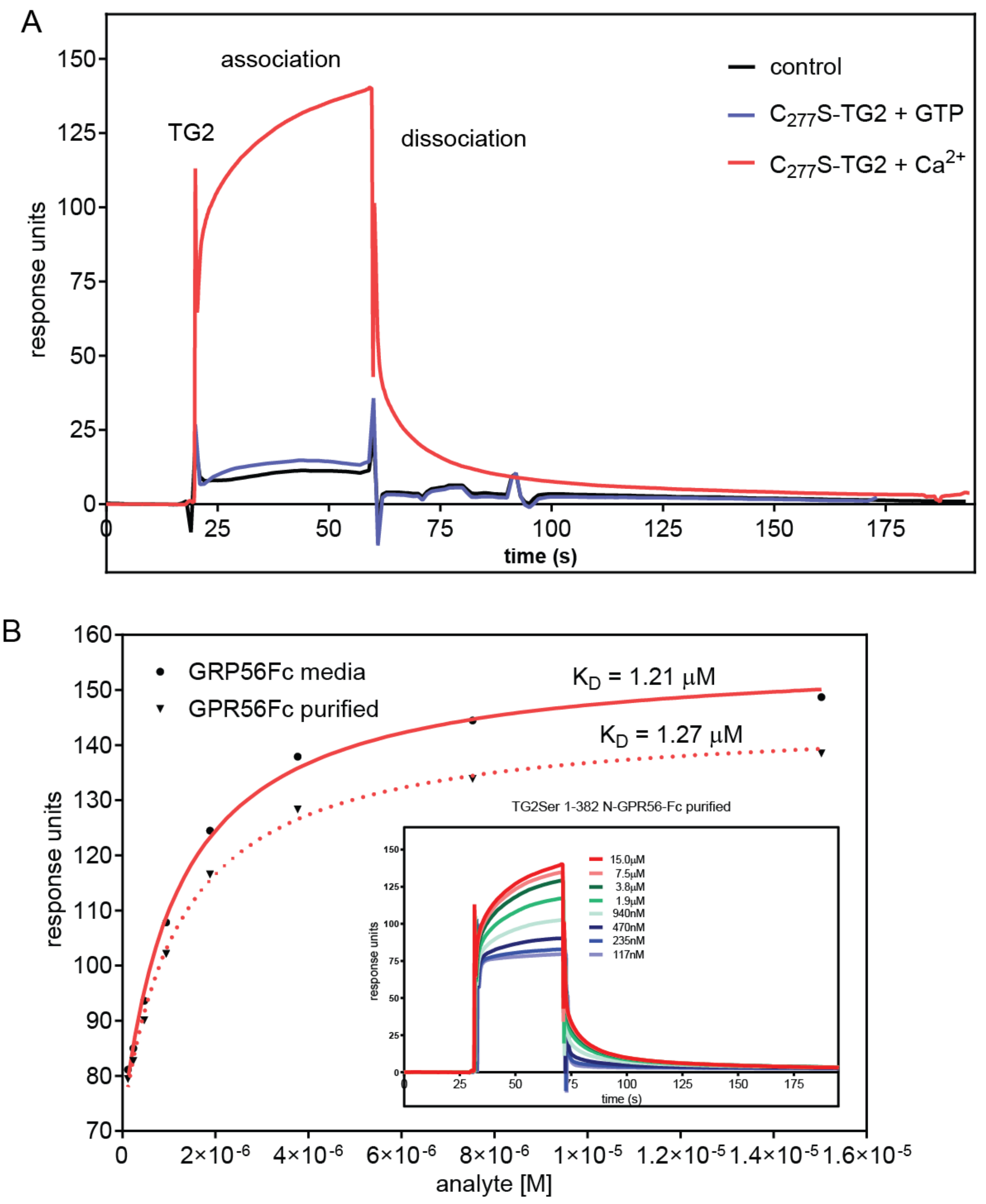

(1, 2)

1. Kielty, C. M., Phillips, J. E., Child, A. H., Pope, F. M., andShuttleworth, C. A. (1994) Fibrillin secretion and microfibril assembly by Marfan dermal fibroblasts Matrix Biol **14**, 191-199 10.1016/0945-053x(94)90008-6
2. Aeschlimann, D., Koeller, M. K., Allen-Hoffmann, B. L., andMosher, D. F. (1998) Isolation of a cDNA encoding a novel member of the transglutaminase gene family from human keratinocytes. Detection and identification of transglutaminase gene products based on reverse transcription-polymerase chain reaction with degenerate primers J Biol Chem **273**, 3452-3460 10.1074/jbc.273.6.3452
